# Antioxidant, pro-oxidant, cytotoxic and antimicrobial properties of selected plant phenolic compounds and resulting structure-activity relationships

**DOI:** 10.1038/s41598-026-50996-z

**Published:** 2026-05-12

**Authors:** Monika Kalinowska, Ewelina Gołębiewska, Małgorzata Zawadzka, Elżbieta Wołejko, Agata Jabłońska-Trypuć, Urszula Wydro, Grzegorz Świderski, Renata Świsłocka, Waldemar Priebe, Włodzimierz Lewandowski

**Affiliations:** 1 Department of Chemistry, Biology and Biotechnology, Institute of Civil Engineering and Energetics, Faculty of Civil Engineering and Environmental Science, Bialystok University of Technology, Wiejska 45E Street, Bialystok, 15-351 Poland; 2 natureTECH Centre for Natural Product Research, Bialystok University of Technology, Wiejska 45E Street, Bialystok, 15-351 Poland; 3 Department of Experimental Therapeutics, The University of Texas MD Anderson Cancer Center, 1901 East Rd., Houston, TX 77054 USA

**Keywords:** Phenolic compounds, Rosmarinic acid, Gallic acid, Tannic acid, Antioxidant, Pro-oxidant, Cytotoxic, Antimicrobial, Biochemistry, Biotechnology, Drug discovery, Microbiology, Plant sciences

## Abstract

This study provides a comprehensive evaluation of the biological properties of six selected phenolic compounds: chlorogenic, caffeic, *p*-coumaric, rosmarinic, gallic and tannic acids. Their antioxidant potential was determined using DPPH, ABTS, FRAP, CUPRAC, lipid peroxidation inhibition and pro-oxidant assays, while antimicrobial activity was tested against *Staphylococcus aureus* and *Candida albicans*. In addition, cytotoxicity toward human skin melanoma A-375 cells and normal fibroblasts was examined, together with the assessment of intracellular ROS, GSH/GSSG ratio, thiol group content, and lipid peroxidation products. The results revealed clear structural dependence of biological activity, with the number and distribution of hydroxyl groups playing a decisive role. Tannic acid exhibited the strongest antioxidant and antimicrobial properties, while gallic and tannic acids showed the most pronounced cytotoxicity toward melanoma cells, accompanied by elevated oxidative stress and redox imbalance, with limited toxicity toward normal fibroblasts. These findings support the potential application of plant-derived phenolic compounds as natural agents with antioxidant, antimicrobial and anticancer activity, making them promising candidates for pharmaceutical and cosmetic use.

**Supplementary Information:**

The online version contains supplementary material available at 10.1038/s41598-026-50996-z.

## Introduction

**Fig. 1:**
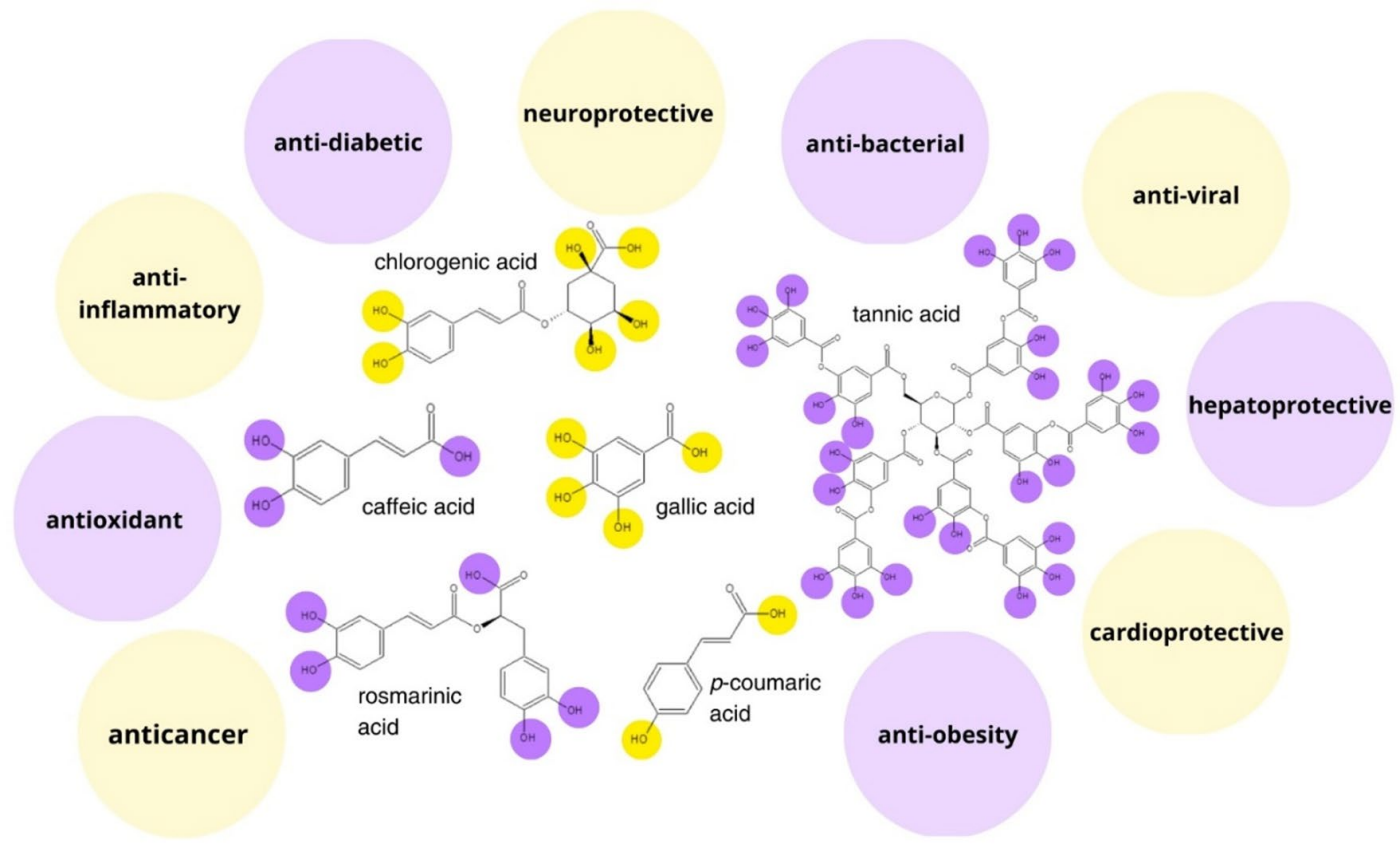
Structures of presented compounds (ACD/ChemSketch, version 2015 Pack 2, Advanced Chemistry Development, Inc., Toronto, ON, Canada, www.acdlabs.com) and their biological activity: **(a)** chlorogenic acid, **(b)** caffeic acid, **(c)** *p*-coumaric acid, **(d)** rosmarinic acid, **(e)** gallic acid, **(f)** tannic acid. Based on^3,7–57^. Created by. Gołębiewska, E., and Zawadzka, M. (2026) via Canva.com.

Recently, a growing interest in plant bioactive compounds and their potential applications in pharmaceuticals and foods (especially functional foods) has been observed. Notably, phenolic compounds, such as phenolic acids and tannins sourced from plants, arouse great interest due to their efficacy in counteracting oxidative stress and promoting human health. They can be found in a wide variety of natural sources such as fruits (e.g. apples, berries, pears, grapes, pomegranate, mango)^1,2^, vegetables (e.g. onion, celery, potatoes)^1^, cereal (e.g. maize, oats and wheat)^3^, herbs (e.g. estragon, parsley, rosemary)^4^, coffee^4^, tea^5^ and cocoa^6^. Detailed information regarding specific sources and extraction methods of these compounds are shown in Table S1. In this study we selected six phenolic compounds with outstanding biological properties were selected, i.e. chlorogenic acid (5-caffeoylquinic acid, 5-CQA), caffeic acid (CA), *p*-coumaric acid (*p*-CA), rosmarinic acid (RA), gallic acid (GA) and tannic acid (TA)^3,7–57^(Fig. 1). TA is a hydrolysable tannin included as a structurally complex reference compound.

The selected phenolic compounds represent promising natural anticancer agents in melanoma therapy. Reports show that 5-CQA exhibits anticancer effect on the melanoma A-375 cell line. Tosoc et al.^58^ studied the activity of 5-CQA from *Corchorus olitorius* on human melanoma (A-375), gastric (AGS), and pancreatic (SUIT-2) cell lines using the MTT (3-(4,5-dimethylthiazol-2-yl)−2,5-diphenyltetrazolium bromide) cell viability assay.

The studies revealed that the application of 30 µM 5-CQA effectively impeded the proliferation of cancer cells following a 48-hour incubation period. Moreover, observations derived from chick chorioallantoic membrane (CAM) models indicated that 5-CQA exhibits properties that counteract angiogenesis and tumor growth in A-375 tumors. Specifically, the administration of 30 µg 5-CQA per CAM significantly diminished the levels of tumor hemoglobin (Hb) over an eight-day treatment duration by impeding the angiogenesis process. Kianmehr et al.^59^ investigated the influence of laser irradiation on anticancer activity of *p*-CA using A375 and SK-MEL-37 cell cultures. The human dermal fibroblast, A-375, and SKMEL-37 cells were exposed to low-level laser at 660-nm wavelength with 3 J/cm^2^ for 90 s, and then the cells were treated with different concentrations of *p*-CA (0–1000 µg/mL for 24 h). Their study demonstrated that prior exposure to low-level laser irradiation followed by treatment with *p*-CA led to a decrease in the survival and proliferation of melanoma cells. In the melanoma A-375 cell line, the toxicity of *p*-CA within the specified concentration range, without laser irradiation, was insignificant up to 100 µg/mL. However, the cytotoxic effect became more pronounced at higher concentrations, reaching approximately 31.6% cell viability at 1 mg/mL of *p*-CA. Based on our results, the half maximal inhibitory concentration (IC_50_) of p-CA for the A-375 melanoma cell line was determined to be approximately 800 µg/mL (4880 µM) following a 24-hour incubation in the dark.

Yoon et al.^60^ found that *p*-CA shows inhibitory effect on melanogenesis induced by α-MSH in B16/F10 murine melanoma cells. It was investigated by incubating melanoma cells with 500 µg/mL of *p*-CA for 48 h. RA was found to regulate different signaling pathways in melanoma, including the protein kinase A/cAMP response element-binding protein/microphthalmia-associated transcription factor (PKA/CREB/MITF) axis, while also acting as a free radical scavenger and detoxification enzyme modifier^61^. Kyriakou et al.^62^ studied anticancer potency of *Salvia fruticosa*, which contains a high amount of RA in a model of human melanoma. The corresponding EC_50_ value was calculated to be 0.048 mg/mL at 48 h post-exposure. Cattaneo et al.^63^ also investigated the anticancer effect of plant extract (*Rosmarinus officinalis* L.) containing RA on human melanoma A-375 cells. Results showed that rosemary extract reduced cell growth; the anti-proliferative effect was evident as early as 24 h and became enhanced after 48 and 72 h of incubation. The IC_50_ estimated after 72 h incubation was 1:480 dilution.

GA exhibits cytotoxic properties against A375.S2 human melanoma cells, inducing apoptosis through both caspase-dependent and caspase-independent pathways^64^. Additionally, various gallates, including octyl, decyl, dodecyl, and tetradecyl gallates, have been found to exert cytotoxic effects on melanoma cells by depleting glutathione levels and subsequently inhibiting γ-glutamylcysteine synthase activity^65^. In addition to its cytotoxic effects, GA also demonstrates other significant biological functions in relation to melanoma cells, such as its antimelanogenic properties^66^ and its ability to inhibit cell adhesion^65^. Liu et al.^64^ studied the antitumor effect of GA on mouse melanoma B16F10 cells. When 400 µM GA was introduced, the assay revealed a considerable induction of cell death, with approximately 40% of cells being affected. Khorsandi et al.^67^ studied anticancer effect of GA on the A-375 cell line. The findings indicated a reduction in the survival of melanoma cancer cells in the presence of GA, with a cell viability of approximately 23% observed at a concentration of 200 µg/mL. Based on the results, the half-maximal inhibitory concentration (IC_50_) of GA on the melanoma A-375 cell line was estimated to be around 100 µg/mL after 24 h of incubation.

Moghaddam et al.^68^ evaluated the effects of polyvinyl alcohol/carboxymethyl cellulose-tannic acid hydrogels (PVA/CMC-TA) on the cell viability of natural human dermal fibroblast (NHDF). The PVA/CMC-TA hydrogels containing 5% of TA did not show toxicity in NHDF cells for up to 72 h. They also investigated a cytotoxicity of the CS-TA/PDI hydrogels (chitosan/tannic acid as an antioxidant cross-linker and loaded with water-soluble, N,N′-di-(L-alanine)−3,4,9,10-perylene tetracarboxylic diimide (PDI-Ala) as a photosensitizer on human melanoma A-375 cells and non-cancerous human dental pulp stem cells (DPSCs) by MTT assay. CS-TA/PDI hydrogels exhibited cytoprotective and antioxidant effects on DPSCs, while A-375 cell viability decreased with higher concentrations of PDI-Ala and TA. Notably, hydrogels containing 40 wt% TA demonstrated the most effective phototoxicity activity, indicating increased sensitivity of A-375 cells to higher TA concentrations in photodynamic therapy. Although A-375 cells exhibited cytotoxicity, no alterations in DPSCs’ cell morphology were observed following light irradiation.

Reports from the last decade regarding the activity of CA against melanoma A-375 are very few. For example, caffeic acid phenethyl ester (CAPE) suppresses the growth of melanoma cells and induces ROS generation^69^. The administration of 10 mg/kg/day of CAPE resulted in significant suppression of B16F0 tumor xenograft growth in C57BL/6 mice. Tumors derived from CAPE-treated mice exhibited reduced phosphorylation of phosphoinositide 3-kinase, AKT, mammalian target of rapamycin, and protein levels of X-linked inhibitor of apoptosis protein (XIAP). Additionally, there was an enhancement in the cleavage of caspase-3 and poly (ADP ribose) polymerase.

In this paper we selected structurally diverse phenolic compounds and screened for their biological properties (anticancer, antimicrobial, antioxidant) under the same experimental conditions. This allowed for a systematic approach to dissect the relationship between the structure of the phenolic compounds and their biological activity. The chosen structures of phenolic compounds differ in the number and position of hydroxyl groups, moreover, their skeleton is a cinnamon or gallic moiety. Therefore, in this paper, we focused on examining the cytotoxicity of the selected phenolic compounds against skin cancer melanoma A-375 cells and normal human skin fibroblasts. To elucidate the mechanism of action of phenolics, we determined the levels of cellular reactive oxygen species (ROS), total protein, and sulfhydryl groups (-SH), as well as the reduced-to-oxidized glutathione ratio (GSH/GSSG) and membrane lipid peroxidation products (measured as TBARS). Because the anti-/pro-oxidant activity of phenolics is one of the mechanism related to their anticancer or even antimicrobial properties, spectrophotometric antioxidant assays including DPPH (2,2-diphenyl-1-picrylhydrazyl), ABTS (2,2’-azino-bis(3-ethylbenzothiazoline-6-sulfonic acid)), FRAP (ferric reducing antioxidant power), CUPRAC (cupric reducing antioxidant power), lipid peroxidation inhibition and pro-oxidant Trolox assays were performed, taking into account the structural diversity of the selected phenolic compounds. The analysis and comparison of antioxidant activity (estimated by the use of five different methods) of six phenolic compounds with key differences in their molecular structure allowed drawing important conclusions regarding the selection and designing of effective antioxidants. Additionally, the antimicrobial properties of the selected phenolics against bacteria *Staphylococcus aureus* and yeast *Candida albicans* were examined to assess the possibility of using them as effective antimicrobial natural agent with potential application in the pharmaceutical or cosmetic industry.

## Materials and methods

### Materials

Chlorogenic acid, caffeic acid, *p*-coumaric acid, rosmarinic acid, gallic acid and tannic acid were provided by Sigma-Aldrich. K_2_S_2_O_8_, ammonium acetate, ABTS, Trolox, acetate buffer, TPZT, FeCl_3_, FeCl_2_, FeSO_4_, horseradish peroxidase, ammonium rhodate and linoleic acid were also obtained from Sigma-Aldrich. DPPH was purchased from TCI. CuCl_2_ · H_2_O was provided by Alfa Aesar. Neocuproine was provided by J&K Scientific. HCl was obtained from Stanlab. Phosphate buffer was purchased from Fluka Analytical. Methanol was provided by POCH basic and ethanol by Chempur. All chemicals were of analytical grade and were used as received without any further purification.

Dulbecco’s modified Eagle’s medium (DMEM) with 4.5 mg/mL (25 mM) of glucose with Glutamax, penicillin, streptomycin, trypsin–EDTA, FBS (Fetal Bovine Serum) Gold, and PBS (Phosphate Buffer Saline) (without Ca and Mg) were provided by Gibco (San Diego, CA, USA). MTT reagent was purchased from Sigma-Aldrich. Dichlorodihydrofluorescein diacetate (DCFH-DA) was obtained from Sigma, St. Louis, MO, USA. GSH/GSSG-Glo kit was obtained from Promega Madison, WI). SDS, TCA, TBA, Folin-Ciocalteu reagent were provided by Sigma-Aldrich and DTNB by Serva.

### Methods

#### UV Studies

The UV/Vis spectra of the studied compounds dissolved in methanol at a concentration of 5∙10^− 5^ M were recorded in the wavelength range of 200–550 nm using a T-9200 spectrophotometer (Peak Instruments, Houston, USA).

### Antioxidant studies

#### DPPH assay

In the DPPH assay, 100 µL of the sample was mixed with 200 µL of a 60 µM DPPH methanolic solution in 96-well plates. Because different compounds were tested, individual concentration ranges were adjusted for each compound in order to accurately determine their IC₅₀ (defined as the concentration required to inhibit 50% of DPPH^•^ radicals) values. Overall, the tested concentration range spanned from 0.33 to 10,000 µM. Absorbance was measured after 60 min at 516 nm using a Tecan Infinite 200 PRO microplate reader (Tecan, Männedorf, Switzerland)^70^. The percentage of DPPH^•^ radical inhibition (%I) was determined using the following formula:

1 $$\:\mathrm{\%}\mathrm{I}=\frac{{A}_{c}\cdot\:{A}_{s}}{{A}_{c}}\cdot\:100\%$$

where: Ac - represents the absorbance of the control sample, As - represents the absorbance of the tested sample. The percentage inhibition values were plotted against compound concentration, and IC₅₀ values were determined from the resulting scavenging curves.

#### ABTS assay

To prepare the ABTS^•+^ cation radical solution, ABTS (5.4 mM) and K_2_S_2_O_8_ (1.74 mM) were combined in equal volumes. The mixture was then incubated in darkness for 12 h. Subsequently, the solution was diluted with methanol to achieve an absorbance in the range of 0.7–0.8 at a wavelength of 734 nm^71^. For the ABTS test, 100 µL of the diluted radical solution was mixed with 100 µL of tested compound solution (the concentration in the sample was 5 µM). Control samples containing methanol instead of the tested compounds were prepared in parallel. A kinetic assay was performed by measuring absorbance at 734 nm every 60 s for 14 min using the Tecan Infinite 200 PRO microplate reader (Tecan, Männedorf, Switzerland). The inhibition percentage was determined using the identical calculation method employed in the DPPH^•^ assay.

#### CUPRAC assay

To perform the CUPRAC assay, 20 µL of the solution of tested compound was mixed with 180 µL of CUPRAC reagent in 96-well plates (the concentration of the tested compounds in the sample was 5 µM). The CUPRAC reagent consisted of 10 mM CuCl2, 7.5 mM neocuproine, and acetate buffer at pH 7 in a ratio of 1:1:1 (v/v/v)^72^. A kinetic assay was performed by measuring absorbance at 450 nm every 10 min for 1 h using the Tecan Infinite 200 PRO microplate reader (Tecan, Männedorf, Switzerland). The antioxidant activity was measured in terms of Trolox equivalents (µM) using a calibration curve generated with Trolox as the standard compound (y = 0.7388x + 0.0639; R²= 0.9985).

#### FRAP assay

In order to determine the Ferric Reducing Antioxidant Power (FRAP), 20 µL of the solution of tested compound was mixed with 180 µL of the FRAP reagent, which consisted of 10 mM, TPTZ, 20 mM FeCl_3_ ·6H_2_O, and 300 mM acetate buffer pH 3.6 in a ratio 1:1:1 (v/v/v)^73^. The concentration of the tested compounds in the sample was 5 µM. A kinetic assay was performed by measuring absorbance at 595 nm every 60 s for 15 min using the Tecan Infinite 200 PRO microplate reader (Tecan, Männedorf, Switzerland). The ferric-reducing antioxidant activity was quantified as Fe(II) ion equivalents (µM) based on the calibration curve obtained using FeSO_4_ (y = 1.1981x + 0.0574; R^2^= 0.9998).

### Pro-oxidant activity

Pro-oxidant activity was determined according to^1^. The concentration of tested compounds in the sample was 5 µM. Measurements were performed using a T-9200 spectrophotometer (Peak Instruments, Houston, USA).

### Lipid peroxidation inhibition assay

The assessment of lipid peroxidation inhibition was estimated by the use of the ferric thiocyanate method as detailed in reference^74^. A linoleic acid emulsion was prepared and mixed with each tested compound solution (the concentration in sample was 0.4 µM). The resulting mixture underwent incubation at 40 °C. Every 24 h over a 5-day period, 0.1 mL of the sample was aliquoted for measurement. The following chemicals were added to each aliquoted sample: methanol, 30% ammonium rhodate solution, and then 0.02 M FeCl_2_ after a 3-minute interval. Subsequently, the absorbance of the sample was promptly measured at a wavelength of λ = 500 nm. A control sample lacking any antioxidant was concurrently prepared. All measurements were conducted with five replicates in three independent experiments. The percentage inhibition of lipid peroxidation was computed using the same formula employed in the DPPH assay. The tested compounds do not absorb in the range of absorption maxima at which the spectroscopic assays for antioxidant activity were performed (Fig. S1).

### Antimicrobial activity assay

Selected model strains *Staphylococcus aureus* (ATCC 25923) and *Candida albicans* (ATCC 10231) were obtained from the American Type Culture Collection (ATCC, Manassas, VA, USA). *S. aureus* (Gram positive bacteria) and *C. albicans* (yeast) were grown overnight in Mueller Hinton II Broth at 37 °C (bacteria strain) and in Sabouraud Dextrose Broth (SDB) at 27 °C (yeast). For the analysis of antimicrobial activity of GA and TA, 10^6^ CFU/mL inoculum of *S. aureus* and 10^4^ CFU/mL inoculum of *C. albicans* cells were used. The antimicrobial activity of tested 5-CQA, CA, *p*-CA, RA, GA and TA against *S. aureus* and *C. albicans* was calculated based on the 3-(4,5-dimethylthiazol-2-yl)−2,5-diphenyltetrazolium bromide (MTT, Sigma-Aldrich) colorimetric assay. The determination of MTT was according to the method reported by Jablonska-Trypuc et al. (2019)^75^ and each treatment was analyzed in triplicate. Furthermore, the Minimum Inhibitory Concentration (MIC) and Minimum Bactericidal/Fungicidal Concentrations (MBC/MFC) were determined according to the methodology described by Balouiri et al.^76^ using the serial two-fold microbroth dilution method. MIC value was evaluated as the lowest concentration of an antimicrobial agent that inhibits the growth or significantly reduces the viability of a microorganism (≥ 90%). In turn, MBC/MFC refers to the minimum concentration necessary to kill bacteria or yeast (> 99.99%), which were determined based on the absence of microbial growth on Mueller-Hinton (MH) agar or Sabouraud Dextrose Agar. Gentamycin (10 µg/mL against *S. aureus*) and itraconazole (10 µg/mL against *C. albicans*) were employed as positive controls.

### Cell culture

The influence of 5-CQA, CA, *p*-CA, RA, GA and TA was studied in melanoma A-375 (ATCC CRL-1619) cell line and normal human skin fibroblasts FN (CRL-1474 ATCC (CCD-25Sk)), which were obtained from American Type Culture Collection (ATCC). A-375 cells and fibroblasts were cultured in DMEM (Gibco) supplemented with 10% FBS (Gibco), penicillin (100 U/mL), and streptomycin (100 µg/mL) at 37 °C in a humified atmosphere of 5% CO_2_ in air. Cells viability of the both cell lines was examined in response to exposure of 0.5 µM, 1 µM, 5 µM, 10 µM, 20 µM, 50 µM, 100 µM, 200 µM, 300 µM, and 500 µM for each studied compound.

### Chemical treatment of cells

5-CQA, CA, *p*-CA, RA, GA and TA were stored in a refrigerator at temperature 4 °C and stock solutions were prepared by dissolving in Tris HCl buffer. Compounds were added to the cultured cells for a final concentration in the range of 0.5 µM to 500 µM. The control cells were incubated without test compounds.

### Cytotoxicity study

The cytotoxicity of the studied compounds was assessed according to the method of Carmichael using 3-(4,5-dimethylthiazol-2-yl)−2,5-diphenyltetrazolium bromide (MTT)^77^.

### Intracellular ROS detection

The level of intracellular reactive oxygen species (ROS) was measured according to Krętowski et al.^78^. All the experiments were done in triplicate.

### Determination of GSH/GSSG

Total glutathione and GSH/GSSG ratio were each assayed in triplicate via GSH/GSSG-Glo kit (Promega Madison, WI) following manufacturer’s instructions. Cells were seeded in white bottom 96-well plates at either 1 × 10^4^ cells/well (fibroblasts) or 5 × 10^3^ cells/well (A-375 cells), allowed to attach, and treated with studied compounds (5-CQA, CA, *p*-CA, RA, GA and TA) at a concentration of 200 µM each for fibroblasts or 300 µM each for A-375 cells and incubated for 24 h. Prior to the assay growth media were removed and cells washed with PBS.

### Total protein content in cells

The determination of protein concentration was performed spectrophotometrically as per Lowry et al. as described previously^79^. All the experiments were performed in triplicate.

### Determination of SH groups

SH-groups were measured using the method of Rice-Evans as described previously^79^. All the experiments were done in triplicate.

### Determination of TBA reactive species (TBARS) levels

The method of Rice-Evans was applied in order to measure the level of membrane lipid-peroxidation products (TBARS), as described previously^79^.

### Statistical analysis

All data are given as mean values ± SD (standard deviation). Differences between the properties of the tested phenolic compounds were determined using one-way ANOVA. In the case of significant differences, means were compared using Tukey’s HSD test at *p* < 0.05. Means obtained for treatments and control in microbial and human cells were compared by Dunnett’s test. Significant differences are represented by *p* ≤ 0.05 (*), *p* ≤ 0.01 (**), *p* ≤ 0.001 (***).

## Results and discussion

### Anti-/pro-oxidant studies

**Fig. 2:**
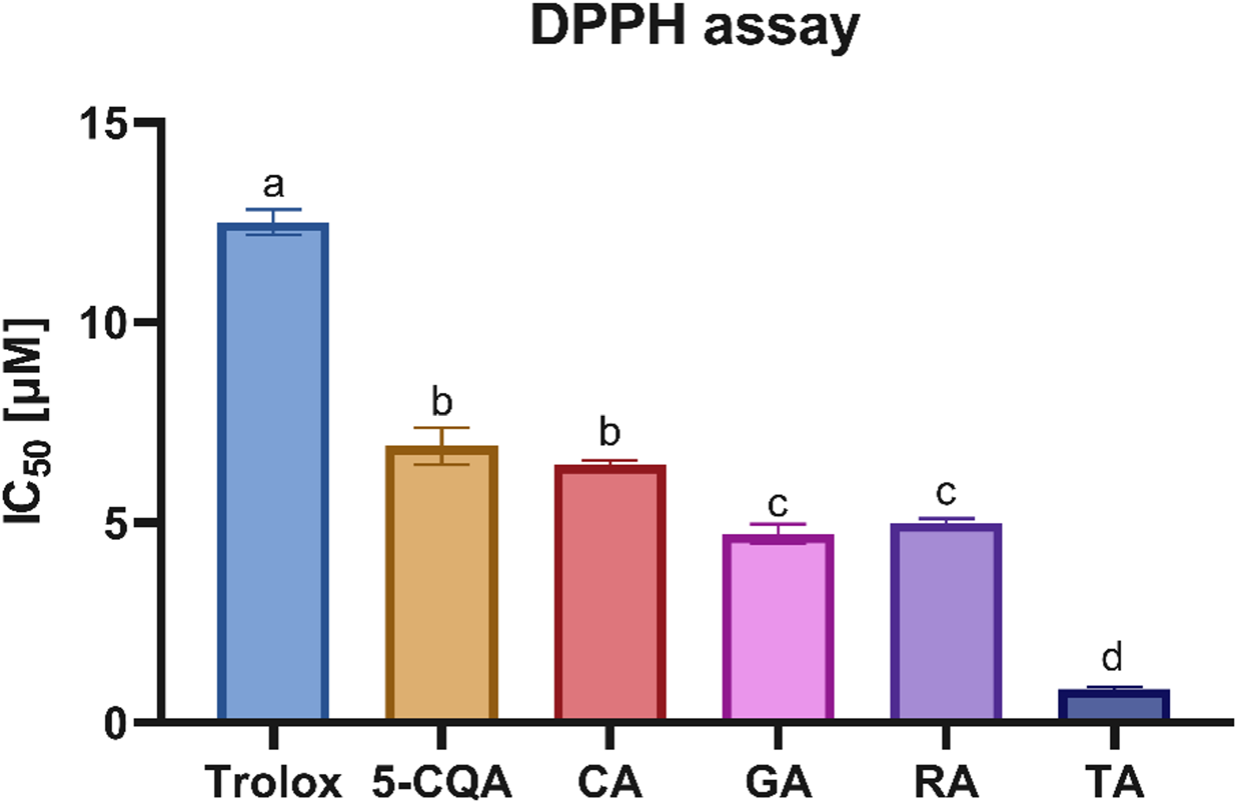
The antioxidant activity of Trolox, 5-CQA, CA, RA, GA and TA determined in the DPPH assay. *p*-CA was excluded from the graphical presentation due to its substantially higher IC₅₀ value (IC₅₀= 13 420.75 ± 664.28 µM). Mean values from three independent experiments ± SD are shown. Different letters above the bars indicate significant differences between means according to Tukey’s post-hoc test at *p* < 0.05.

The antioxidant activity of the six tested phenolic compounds (5-CQA, CA, *p*-CA, RA, GA, TA) was evaluated in DPPH, ABTS, FRAP, CUPRAC and lipid peroxidation assays. In addition, the pro-oxidant activity of the compounds was examined. The DPPH assay stands out as the primary choice for gauging the antioxidant capacity of compounds. Its prominence arises from the robust stability it offers when accepting electrons or hydrogen radicals, making it an excellent choice for assessing antioxidants^80^. In the DPPH assay, significant differences in antiradical activity were observed among the tested compounds (Fig. 2). Trolox was used as the reference antioxidant standard and exhibited an IC₅₀ value of 12.50 ± 0.32 µM. Among the analyzed phenolic compounds, TA (a hydrolysable tannin) exhibited the strongest antiradical activity, with the lowest IC_50_ value (0.85 ± 0.05 µM), indicating markedly higher activity than Trolox. RA (IC_50_= 5.00 ± 0.11 µM) and GA (IC_50_= 4.72 ± 0.25 µM) showed comparable antiradical activity. 5-CQA (IC_50_= 6.92 ± 0.47 µM) and CA (IC_50_= 6.46 ± 0.11 µM) exhibited intermediate activity. In contrast, *p*-CA displayed markedly lower antioxidant activity (IC_50_= 13 420.75 ± 664.28 µM) and was therefore excluded from the graphical presentation due to its substantially higher IC₅₀ value. Based on the IC₅₀ values, the tested compounds can be ranked in ascending order of antioxidant activity (from lowest to highest activity) as follows: *p*-CA < Trolox < 5-CQA < CA < RA ≤ GA < TA.

**Fig. 3:**
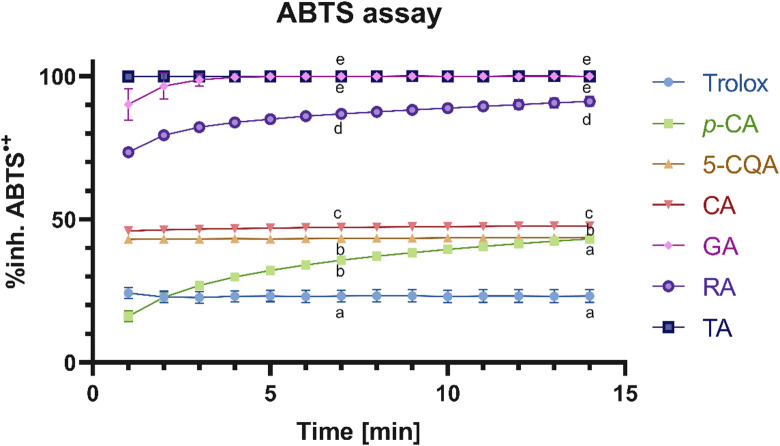
The antioxidant activity of Trolox, 5-CQA, CA, *p*-CA, RA, GA and TA determined in the ABTS assay in time 1–14 min (concentration of all tested compounds was C = 5 µM). Mean values from three independent experiments ± SD are shown. Different letters above the bars indicate significant differences between means according to Tukey’s post-hoc test at *p* < 0.05.

In the ABTS assay, the antioxidants reduce the freshly prepared 2,2-azino-bis(3-ethylbenzothiazoline-6-sulfonic acid) cation radical (ABTS^●+^), leading to a color change from blue-green to colorless and a decrease in absorbance measured at λ = 734 nm^81^. The results of ABTS assay (Fig. 3) displayed a similar trend of compound activity as the DPPH assay (Fig. 2). The percentage of ABTS^●+^ cation radical inhibition by the tested compounds increased over time (Fig. 3). TA and GA at the concentration 5 µM almost entirely inhibited ABTS^●+^ cation radicals within a few minutes (in the examined concentration ranges). RA displayed similar antioxidant activity to TA and GA, demonstrating a 86.85 ± 1.43% inhibition of the ABTS^●+^ cation radicals after 7 min and a 91.25 ± 1.67% inhibition after 14 min. After 7 min, differences in the ABTS^●+^ cation radical inhibition percentages between *p*-CA (%inh.= 35.77 ± 1.21%) and 5-CQA (%inh.= 43.35 ± 0.93%) became noticeable, but after 14 min, these differences were negligible (%inh.= 43.20 ± 1.11% for *p*-CA and %inh.= 43.70 ± 0.95% for 5-CQA). The reference standard, Trolox showed lower inhibition percentages at both evaluated time points (7 min: %inh.= 23.21 ± 2.06% and 14 min: %inh.= 23.19 ± 2.24%) compared to the other tested compounds, indicating weaker radical scavenging activity under the same experimental conditions.

**Fig. 4:**
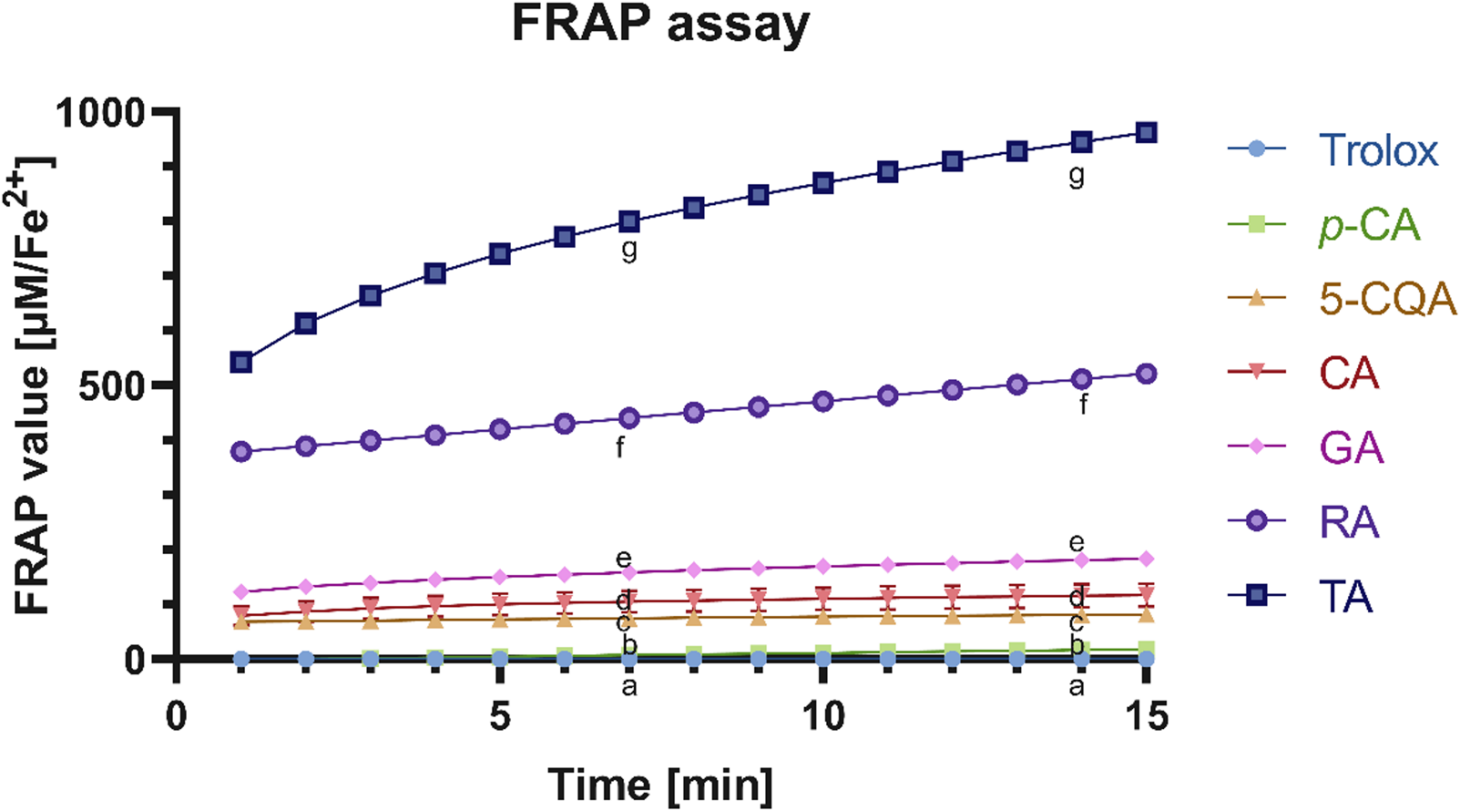
The antioxidant activity of Trolox, 5-CQA, CA, *p*-CA, RA, GA and TA determined in the FRAP assay in time range 1–15 min (concentration of all tested compounds was C = 5 µM). Mean values from three independent experiments ± SD are shown. Different letters above the bars indicate significant differences between means according to Tukey’s post-hoc test at *p* < 0.05.

The term “reducing power” denotes the capacity of atoms, molecules, or ions to release electrons during a chemical reaction. It serves as a crucial indicator of potential antioxidant activity, primarily by assessing the ability of compounds to counteract oxidative processes by donating electrons. This parameter is commonly measured through assays that evaluate the compounds capability to reduce Fe(III) ions to Fe(II) ions (FRAP assay). The increase in reducing power over time reflects the compound’s effectiveness in neutralizing free radicals and protecting biological systems from oxidative damage^82,83^. In the conducted FRAP assay (Fig. 4), the tested compounds at the concentration 5 µM exhibited reducing activity within the range of 7.52–799.39.52.39 µM/Fe^2+^ at a reaction time of 7 min and 16.80–944.89.80.89 µM/Fe^2+^ for a reaction time of 14 min (based on the obtained FeSO_4_ calibration curve). Among them, *p*-CA displayed the weakest reducing activity. 5-CQA demonstrated lower activity than CA. TA exhibited approximately twice the reducing activity compared to RA. The tested compounds can be ranked on the basis of their increasing iron(III) ion reducing activity in the following order: Trolox < *p*-CA < 5-CQA < CA < GA < RA < TA.

**Fig. 5:**
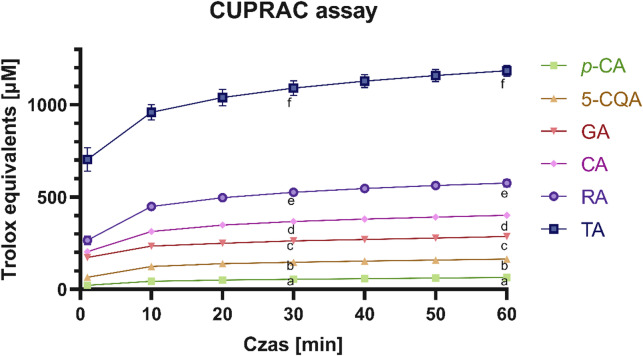
The antioxidant activity of 5-CQA, CA, *p*-CA, RA, GA and TA determined in the CUPRAC assay in time range 1–60 min (concentration of all tested compounds was C = 5 µM). Mean values from three independent experiments ± SD are shown. Different letters above the bars indicate significant differences between means according to Tukey’s post-hoc test at *p* < 0.05.

The CUPRAC assay relies on the conversion of Cu(II) ions into Cu(I) ions due to the presence of non-enzymatic antioxidants in the sample^84,85^. Based on the prepared Trolox calibration curve, it was observed that the tested compounds at the concentration 5 µM exhibited the ability to reduce Cu(II) ions in the range of 65.15–1184.79.15.79 µM of Trolox, depending on the reaction time (Fig. 5). Based on the demonstrated reducing antioxidant capacity, they can be arranged in the following order: *p*-CA < 5-CQA < GA < CA < RA < TA. TA exhibited a capability to reduce Cu(II) ions (1090.59–1184.79.59.79 µM of Trolox) that was twice as potent as RA (526.06–576.09.06.09 µM of Trolox). Furthermore, a minor but noticeable increase in antioxidant activity over time was observed.

**Fig. 6:**
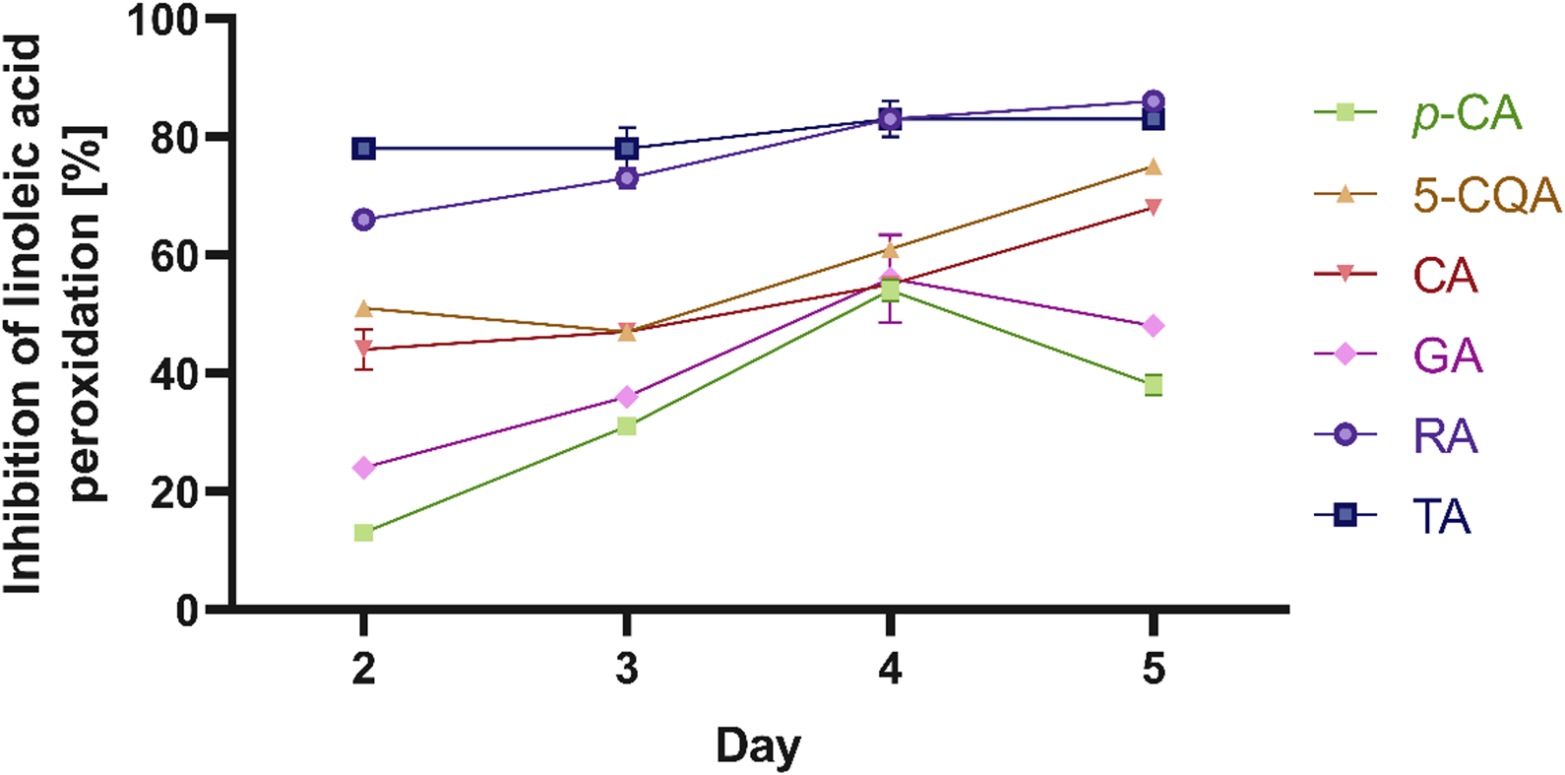
Linoleic acid peroxidation inhibition assay of 5-CQA, CA, *p*-CA, RA, GA and TA (concentration of all tested compounds was 0.4 µM) over the course of 5 days. Mean values from three independent experiments ± SDs are shown.

The lipid peroxidation inhibition assay relies on the reaction of Fe(II) ions with the by-products generated during the peroxidation of linoleic acid. This interaction results in the creation of a red complex involving Fe(III) and thiocyanate ions, which can be detected at a wavelength of 500 nm^86^. TA (78.34 ± 0,45%−83.48 ± 0,57%) and RA (65.55 ± 0,73–85.58.58 ± 0,14%) at the concentration 0.4 µM exhibited the most significant inhibition of lipid peroxidation. These results were comparable to the reference antioxidant Trolox (tested at the same concentration), which showed activity in the range of 30 ± 0.95% to 82 ± 0.95% over the 5 days, reaching 77 ± 1.02% on the 4th day and 82 ± 0.95% on the 5th day. In comparison to the other studied compounds, it is clearly visible that TA exhibited a high and constant potential for inhibiting membrane lipid oxidation over the course of 5 days of experiments. It can be explained by the high lipophilic character of TA (logP = 4.84) compared to RA (logP = 1.60), *p*-CA (logP = 1.79), CA (logP = 1.12), 5-CQA (logP = 0.30), GA (logP = 0.70)^87^. The rest of studies compounds displayed as well inhibitory effect on lipid peroxidation which increased over the days of the experiment (Fig. 6). In comparison to other tested compounds, *p*-CA and GA displayed the lowest inhibition of lipid peroxidation (in the range from 12.55 ± 0,64% to 55.83 ± 7,49%). *p*-CA showed the lowest antioxidant activity among the tested compounds in the above discussed assays as well, therefore it is not surprising that the lowest activity of *p*-CA is also in relation to the inhibition of the degree of lipid peroxidation. In the case of GA, however, high antioxidant activity was observed in earlier tests. Most likely due to the very low lipophilicity of GA and its limited solubility in lipids, GA showed low antioxidant potential in the lipid peroxidation inhibition test. The results obtained for *p*-CA, GA, CA, and 5-CQA on day 4th of measurement were similar (ranging from 54.38 ± 1,76% to 60.72 ± 1,16%) which was notably lower than the activity observed for Trolox (77 ± 1.02%) on the same day. Based on the data from the final day of measurement (5th day), the compound activity can be ranked in ascending order as follows: *p*-CA < GA < CA < 5-CQA < TA ≤ RA.

**Fig. 7:**
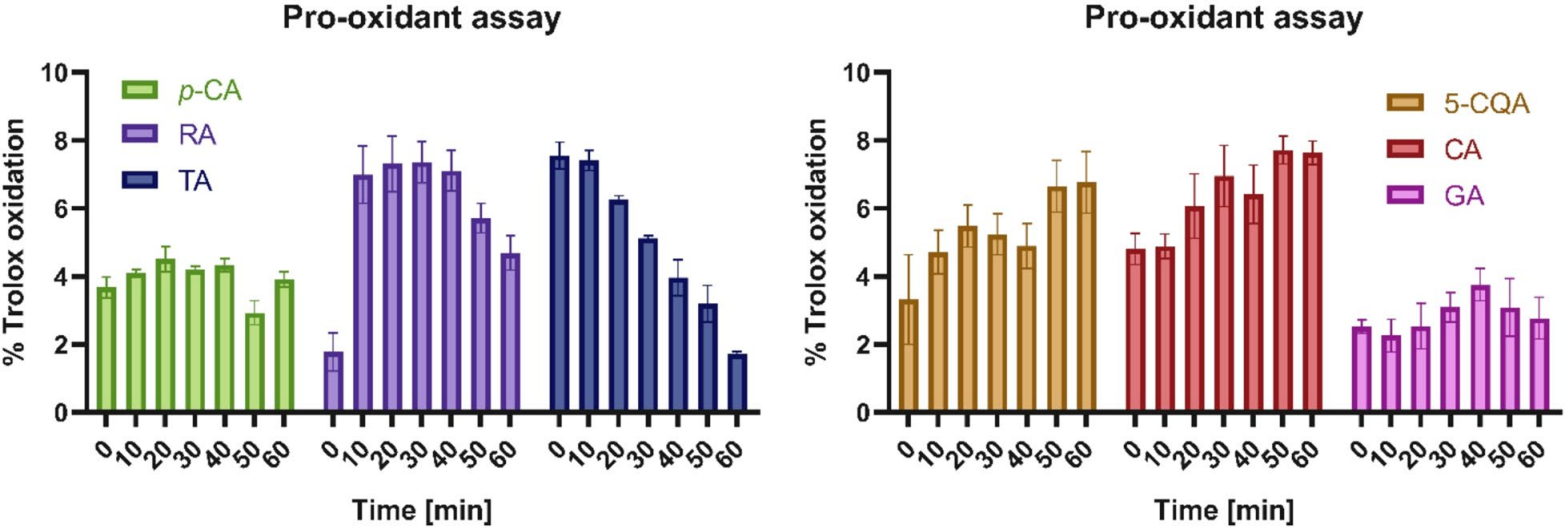
Pro-oxidation activity assay of 5-CQA, CA, *p*-CA, RA, GA and TA (concentration of all tested compounds was C = 5 µM). Mean values from three independent experiments ± SDs are shown.

The assessment of pro-oxidant activity involved evaluating Trolox oxidation rates. In this method, phenolic compound radicals were generated through their interaction with H_2_O_2_, facilitated by horseradish peroxide. Subsequently, these phenoxyl radicals reacted with Trolox, leading to its oxidation into Trolox radicals and further into Trolox quinones. This process transformed the phenoxyl radical into phenolic compounds. The evaluation included measuring the maximum absorption for Trolox quinone at 272 nm^88^. The results indicated the degree of Trolox oxidation ranging from 1.72% to 7.64% for the tested compounds (Fig. 7) at a concentration of 5 µM (and a specific concentration of the remaining reagents described in the experimental section). This suggests that the compounds exhibit limited pro-oxidative potential. At the beginning of the experiment (the first 10–20 min of the experiment), RA and TA showed the highest pro-oxidant properties among the tested phenolic compounds. However, these properties decreased with the passage of time. In the case of 5-CQA and CA, an opposite trend was observed. Their pro-oxidant activity notably increased with the progression of the reaction time. The previous experiment showed that RA and TA possessed very high antioxidant potential and excellent ability to react with radicals, cation radicals and reduce metal ions (like Fe(III) and Cu(II)), higher than 5-CQA and CA. The conducted experiments revealed distinct profiles of changes in the pro-oxidant activity of the analyzed phenolic compounds. This result can be explained by the very high antioxidant potential of RA and TA, which in the early phase rapidly react with free radicals and transition metal ions, generating a considerable amount of reactive phenoxyl intermediates. With time, however, these intermediates undergo stabilization (e.g., through quinone formation), which reduces their further pro-oxidant reactivity. An opposite trend was noted for 5-CQA and CA, whose pro-oxidant activity increased progressively during the experiment. Initially, their contribution to oxidation processes was limited, but the phenoxyl radicals formed in these reactions were relatively long-lived and capable of undergoing secondary redox reactions, which translated into a gradual increase in Trolox oxidation. In contrast, *p*-CA and GA maintained a relatively constant pro-oxidant activity throughout the experimental period. In the case of *p*-CA, this can be attributed to its simple structure and the low reactivity of its radicals, whereas for GA it likely results from the balance between its strong radical-scavenging ability and the stabilization of gallic-derived phenoxyl radicals. These findings indicate that the pro-oxidant profiles of phenolic compounds are closely related to their chemical structure and the dynamics of intermediate stabilization, which is consistent with trends reported in the literature.

Summarizing the obtained results of antioxidant activity, it can be concluded that among the six tested phenolic compounds, TA shows the greatest antioxidant properties, followed by RA and GA. The stronger activity of TA is attributed to its polyphenolic structure and high hydroxyl density. The antioxidant potential of phenolic compounds is closely related to the number and location of hydroxyl (-OH) groups on the aromatic ring, their mutual position, and the type of substituents present^89–91^. TA, classified as a hydrolysable tannin, comprises a central glucose molecule and ten galloyl groups. This extensive presence of phenolic hydroxyl groups in the TA structure is responsible for its strong antioxidant properties^92^. The connection between the number of hydroxyl groups within the aromatic ring and antioxidant activity is well-documented^93–95^. This trend remained consistent across assays, such as DPPH, ABTS, and FRAP. *p*-CA, featuring a single -OH group, consistently exhibited lower antioxidant activity when compared to CA with two -OH groups and GA containing three -OH groups. This underscores the significant influence of hydroxyl group quantity on antioxidant potential. In the conducted assays (DPPH, ABTS, CUPRAC, and FRAP), 5-CQA exhibited weaker antioxidant properties compared to CA. The structural differences between 5-CQA, an ester of caffeic acid with quinic acid, and CA are essential to understanding this distinction. The complex structure of 5-CQA, combined with the esterification of CA by a sugar moiety, is correlated with a reduced antioxidative activity, as previously reported by Cuvelier^96^. Accordingly, the complex structure of 5-CQA limits its ability to effectively donate hydrogen atoms or electrons to free radicals, diminishing its antioxidant effectiveness. In contrast, CA features multiple free hydroxyl groups directly on its aromatic ring, enhancing its ability to engage in electron transfer and radical scavenging reactions. This structural simplicity enables CA to more effectively neutralize various radicals, such as DPPH^●^ or ABTS^●+^ radicals, resulting in superior antioxidant performance in the aforementioned assays. A similar relationship was observed in the work of other authors^90,97^.

In our previous publications, we showed that the antioxidant properties of ligands are not only determined by the number of hydroxyl and methoxy groups and their position within the aromatic ring. The number of hydroxyl groups is the most important factor, but other effects also play a role. The potency and effectiveness of antioxidants and their ability to dissociate protons or electrons are also influenced by other factors, such as: (a) the electronic charge distribution in the ligand, (b) the length of conjugated double bonds, and (c) the energy differences between the molecules or between the free radical and the antioxidant. In our opinion, the current research has led to very interesting conclusions and confirmed our previous hypotheses. Tannic acid possesses the strongest antioxidant and reducing properties among the ligands studied. This was demonstrated by five independent methods of measuring antioxidant properties (DPPH, ABTS, FRAP, CUPRAC, and lipid peroxidation assay). Notably, in the lipid peroxidation inhibitory assay, the activity of TA and RA was comparable to that of the reference standard Trolox, underscoring their significant potential in protecting biological lipid systems. TA contains the largest number of hydroxyl groups, but also a very long system of conjugated double bonds and many aromatic rings with delocalized bonds. TA also possesses a large number of quasi-aromatic hydrogen bond systems and strong intra- and intermolecular interactions that lower the energy of this ligand. This also influences the effectiveness of this antioxidant and its reducing power. RA also has very strong antioxidant properties, with a large number of hydroxyl groups but also an extensive system of conjugated double bonds and two aromatic rings. The properties of hydroxyl groups and the potential for proton or electron dissociation are significantly influenced by the rest of the molecule, the degree of electronic charge delocalization, and the ability to form intramolecular and intermolecular hydrogen bonds. Therefore, the relationship between molecular structure and biological activity is not straightforward, and the final effect depends on various interrelated factors.

### Cytotoxic activity

**Fig. 8:**
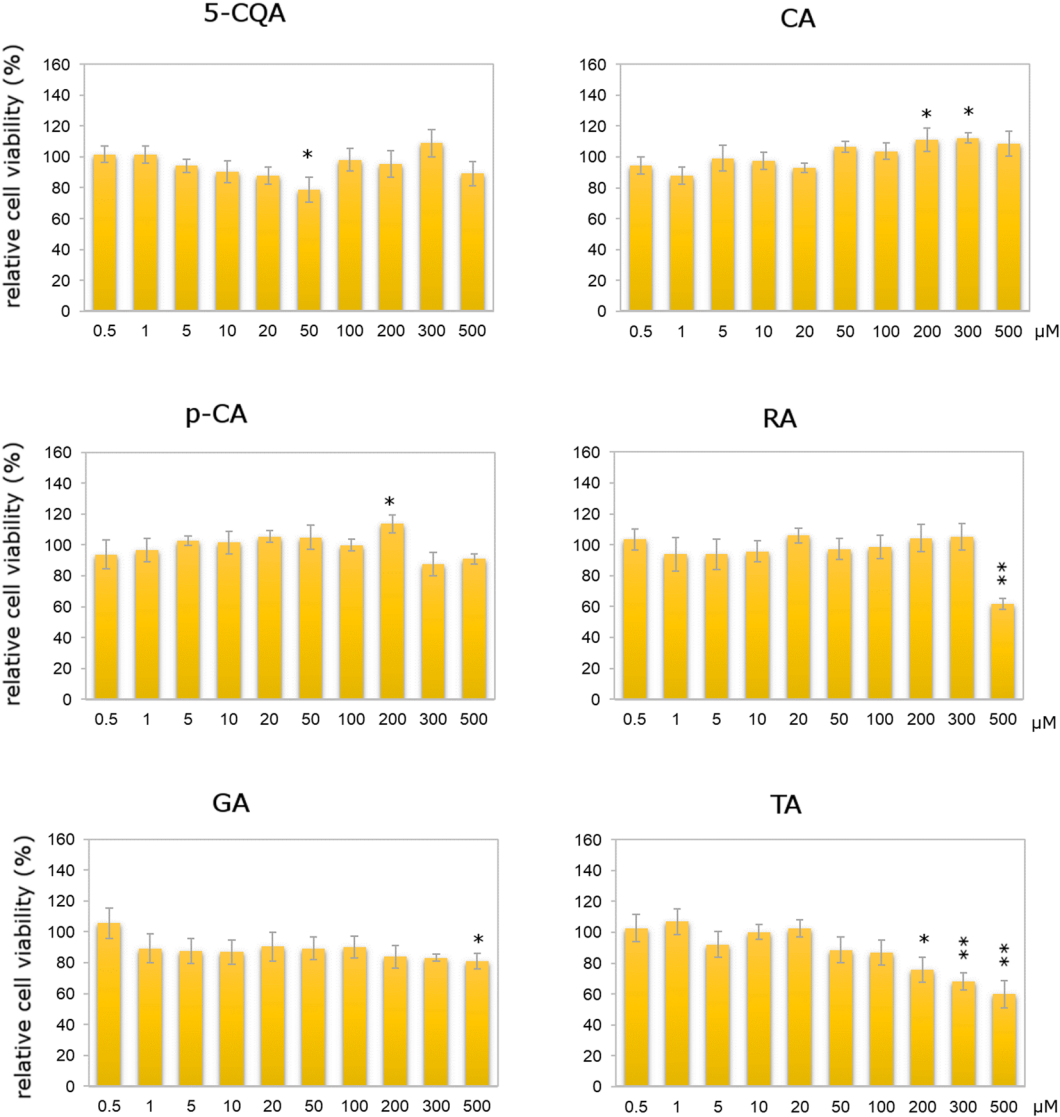
Cell viability results for fibroblasts exposed to different concentrations of 5-CQA, CA, *p*-CA, RA, GA and TA for 24 h calculated as a percentage of control untreated cells. Each value on the graph is the mean of three independent experiments and error bars show the standard deviation (SD); * *p* < 0.05, ** *p* < 0.01, and *** *p* < 0.001 represent significant effects between treatments and control followed by Dunnett’s test.

**Fig. 9:**
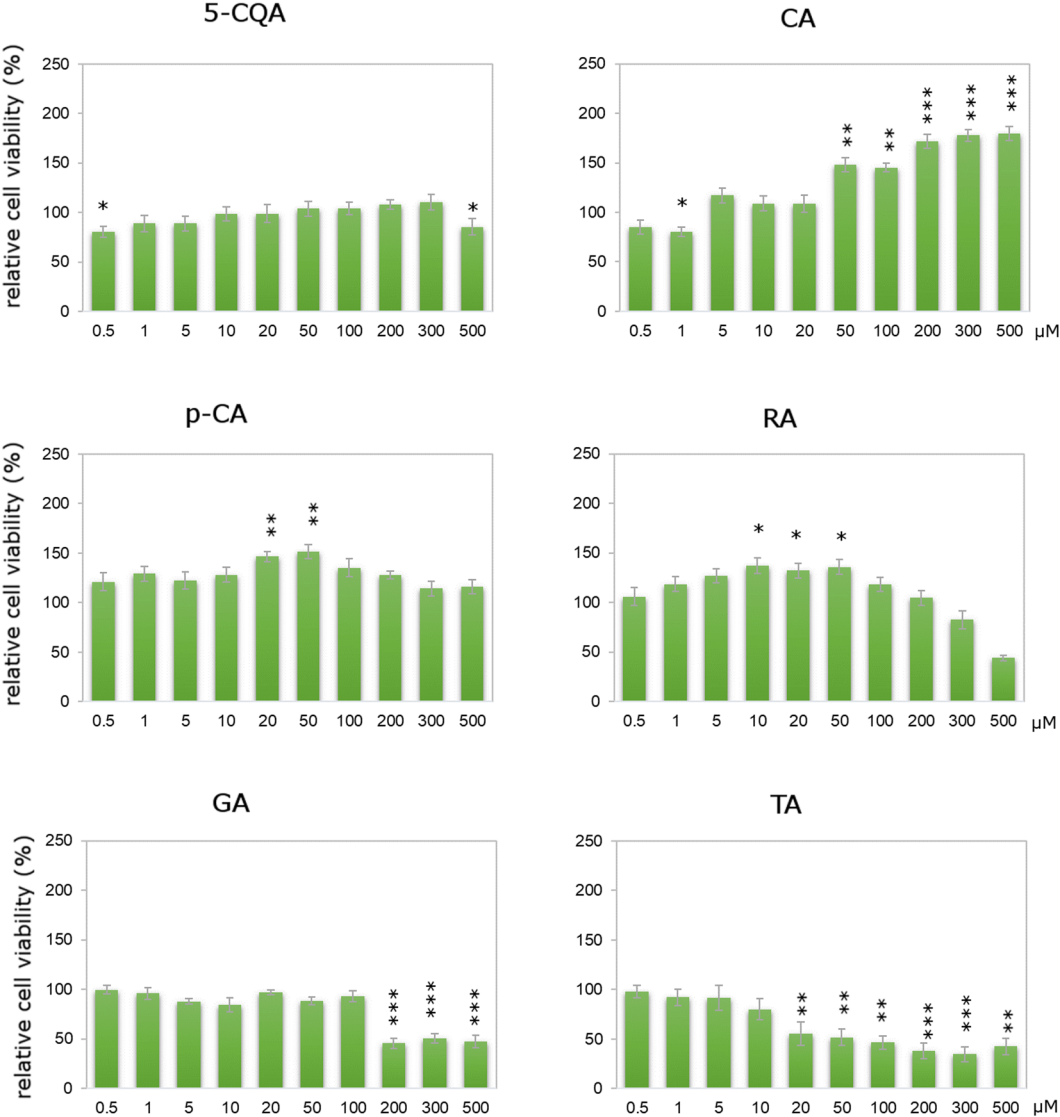
Cell viability results for A-375 cells exposed to different concentrations of 5-CQA, CA, *p*-CA, RA, GA and TA for 24 h calculated as a percentage of control untreated cells. Each value on the graph is the mean of three independent experiments and error bars show the standard deviation (SD); * *p* < 0.05, ** *p* < 0.01, and *** *p* < 0.001 represent significant effects between treatments and control followed by a Dunnett’s test.

The cytotoxicity of 5-CQA, CA, *p*-CA, RA, GA and TA (0.5 µM – 500 µM) after 24 h of incubation with cancer and normal cell lines using the MTT assay was evaluated. The analyzed compounds caused different effects in fibroblasts relative viability (Fig. 8), which was noticed after 24 h treatment. RA exhibited a rather inhibitory effect; for example, 500 µM RA caused approximately 60% inhibition in cell proliferation. On the other hand, 200–500 µM TA inhibited growth of fibroblast by 80 − 60% depending on the concentration. In the case of *p*-CA, no significant effect was observed. Only the highest studied concentration (500 µM), caused a decrease by about 10% in cell viability. The other analyzed phenolic compound, CA, caused no significant decreases below the control level, however some increases in relative cell viability were observed. The highest increase in fibroblast cell proliferation was noticed in response to 300 µM CA. When analyzing A-375 cells (Fig. 9), different concentration-dependent effects were observed. 5-CQA at the lower concentrations decreased A-375 cell viability, which is consistent with literature data regarding its influence on melanoma cells. Kimsa-Dudek et al. found out that 5-CQA in C32 melanoma cells suppressed the viability of melanoma cells^98^. At higher CA concentrations, increased relative cell viability versus control untreated cells was observed. However, lower concentrations of CA caused decreases in relative cell viability, similar to findings reported by Yang et al., who revealed that CA suppresses colony formation in human A431 skin cancer cells^99^. *p*-CA, increased cell viability after 24 h treatment, which is opposite to the data obtained by Tehami et al. indicating anticancer effects of *p*-CA[33]. RA exhibited significantly stimulatory effect on A-375 cell viability, with the exception of the 200–500 µM concentration. The strongest inhibitory effect we detected was due to TA treatment, which 20 µM (and higher until 500 µM) inhibited growth of A-375 cells by ~ 50%. The same inhibitory effect was observed in the case of GA at the range of concentration 200–500 µM.

**Fig. 10:**
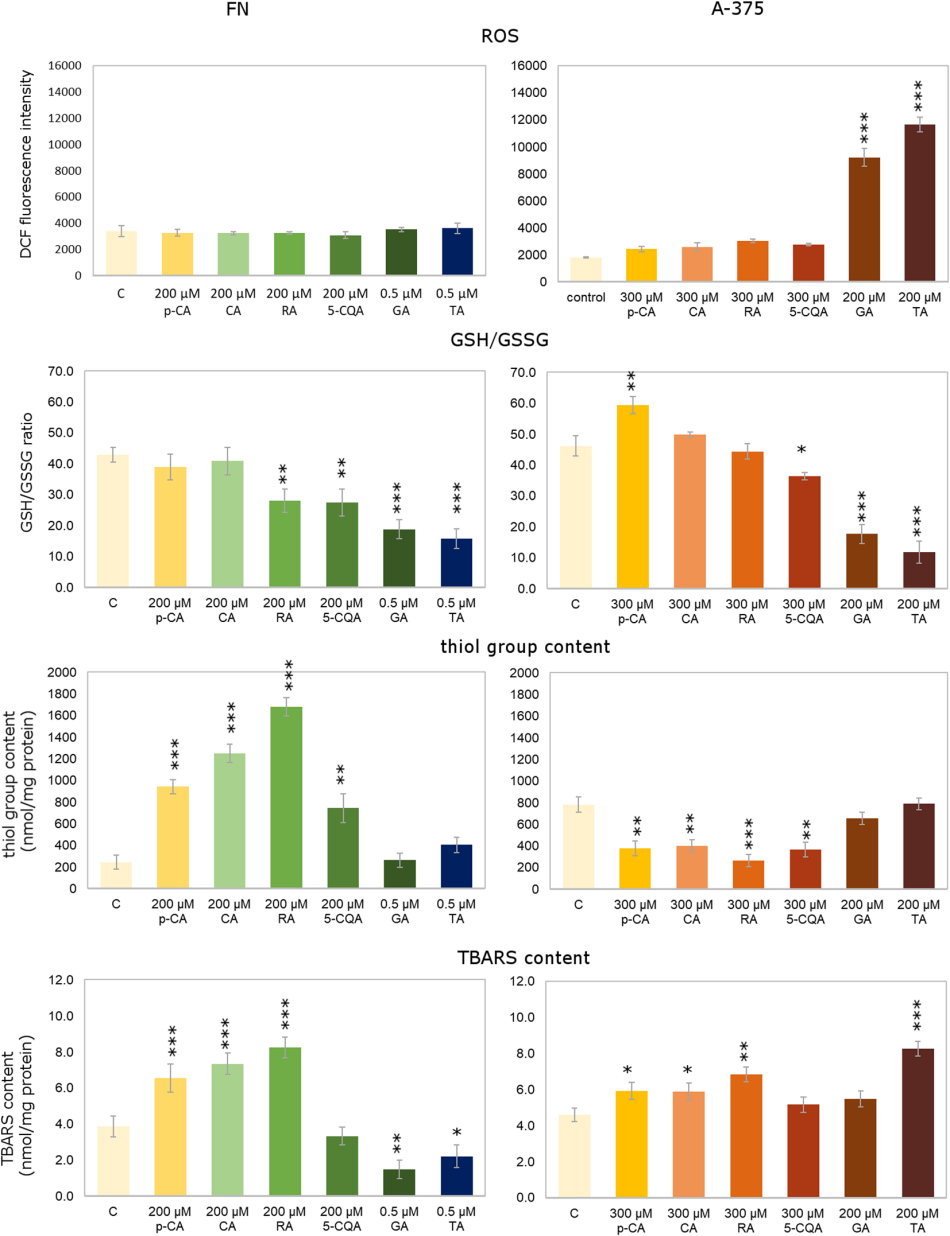
The effect of 5-CQA, CA, *p*-CA, RA, GA and TA on the level of intracellular ROS, GSH/GSSG ratio, thiol groups and TBARS content in fibroblasts and A-375 cells after 24 h of incubation. Each value on the graph is the mean of three independent experiments and error bars show the standard deviation (SD); * *p* < 0.05, ** *p* < 0.01, and *** *p* < 0.001 represent significant effects between treatments and control followed by Dunnett’s test.

Figure 10 shows the influence of studied phenolic compounds on the production of ROS in fibroblasts and A-375 cells. The relative ROS amount is presented as the intensity of fluorescence of 2,7-DCF in both cell lines cultured with *p*-CA, CA, RA and 5-CQA for 24 h. Incubation of fibroblasts with all compounds (C = 200 µM) did not affect the ROS content, and in the case of melanoma cells (compounds concentration 300 µM), an increase in ROS production was observed. The influence of *p*-CA, CA, RA and 5-CQA on GSH/GSSG ratio in fibroblasts and melanoma cells is also depicted in Fig. 10. Our results are similar to those obtained by Balupillai et al., who revealed that CA effectively reduces the generation of ROS, and decreases the frequency of apoptosis in human dermal fibroblasts^100^. The main role of GSH as one of the most important natural antioxidants is to maintain oxidative balance in cells. At a concentration of 200 µM, both RA and 5-CQA significantly decreased GSH/GSSG ratio after 24 h incubation in fibroblasts, while 300 µM of *p*-CA and CA increased the tested parameter in melanoma cells as compared to control. Additionally, changes in thiol group content in both cell lines under the influence of phenolic compounds are presented in Fig. 10. A statistically significant increase in SH group content was detected due to exposure of all studied compounds (C = 200 µM) in fibroblast cells. Alternatively, a decrease was observed in SH group content of melanoma cells (C = 300 µM). In both cell lines TBARS content was elevated with the exception of 200 µM 5-CQA treatment in fibroblasts.

As illustrated in Fig. 9 in case of melanoma cells significant toxicity of GA and TA was observed at the concentrations of 200–500 µM and 20–500 µM, respectively. This is in agreement with literature data indicating the ability of GA to induce apoptosis in A375.S2 human melanoma cells^101^. In fibroblasts (Fig. 8), GA and TA exhibited low toxicity at the lowest concentration (0.5–5 µM). Figure 10 shows the relative fluorescence intensity of 2′,7′-dichlorofluorescein (DCF) as a percent of control in fibroblast cells incubated with 0.5 µM of GA and TA for 24 h and in melanoma cells incubated with 200 µM of GA and TA for 24 h. An increase in the intracellular ROS production resulted in higher intensity of DCF fluorescence and it was observed in both studied cell lines – normal and cancerous. After 24 h incubation of melanoma cells with 200 µM TA, the intracellular ROS generation was significantly higher as compared to control, untreated cells. GA and TA (C = 0.5 µM) treatment caused non-significant increases in intracellular ROS production in fibroblast cells. The results show stimulatory effect of these compounds (C = 200 µM) on ROS content in melanoma cells, which can be linked to an increase in oxidative stress level. As compared to normal cells, cancer cells are characterized by high levels of ROS, which may result from genetic disorders causing uncontrolled proliferation. The compounds tested showed significant antiproliferative properties in cancer cells at the concentration 200 µM. Cancer cell proliferation is inextricably linked to oxidative stress. Our results demonstrate a significant increase in ROS levels in A-375 cells treated with GA and TA, which may be related to the antiproliferative activity of the tested compounds. Determining the GSH/GSSG ratio is a very important parameter in oxidative stress research, because reduced glutathione is one of the most important low-molecular antioxidants. GA in 0.5 µM concentration treatment caused a significant decrease in GSH/GSSG ratio after 24 h of incubation, while 0.5 µM of TA treatment resulted in significant increases in fibroblast cells versus controls. A-375 cell treatment with GA at 200 µM concentration resulted in a significantly elevated ratio of GSH/GSSG. After 24 h of A-375 cells exposure to TA the level of GSH was also elevated as compared to control untreated cells. These results revealed a stimulatory influence of the tested compounds in melanoma cells and inhibitory effect of GA on GSH amount in the fibroblast cell line. The effect of both phenolic compounds on SH group content is presented in Fig. 10. In order to study the oxidation level of the SH group, a spectrophotometric assay with Ellman’s reagent was applied. A significant increase in thiol group content of ~ 65% compared to the control was observed especially at a concentration of 0.5 µM TA in fibroblast cells. Exposure to 200 µM of GA resulted in ~ 17% decrease in the total cellular content of the thiol groups in melanoma cells. The increase in the level of SH groups observed in fibroblasts may indicate an increase in their resistance to oxidative stress and other types of cell damage and also positively correlates with increased cell proliferation, which was observed. In the A-375 cell line, the observed decrease in the level of SH groups correlates with an increase in the level of TBARS in the case of GA and indicates an increased level of oxidative stress with a simultaneous observed decrease in the level of cancer cell proliferation. The level of intracellular TBARS was measured as an indicator of the lipid peroxidation process. This process is associated with various intracellular dysfunctions, which often result from inappropriate modifications of complex lipid-protein macromolecules. The results showed significant differences between TBARS levels in fibroblast cells and melanoma cells depending on compound concentration (Fig. 10). The addition of GA in 0.5 µM to the fibroblast cells induced a significant decrease in TBARS content of ~ 62% compared to the control after 24 h treatment. GA at a concentration of 200 µM induced an increase of ~ 155% compared to the control observed after 24 h of incubation. The obtained results suggest that the studied compounds (C = 0.5 µM) demonstrate protective properties against TBARS production in fibroblasts by decreasing membrane phospholipid peroxidation, but they exhibit pro-oxidative properties (200 µM) in A-375 cells by increasing the peroxidation process.

### Antimicrobial studies

**Fig. 11:**
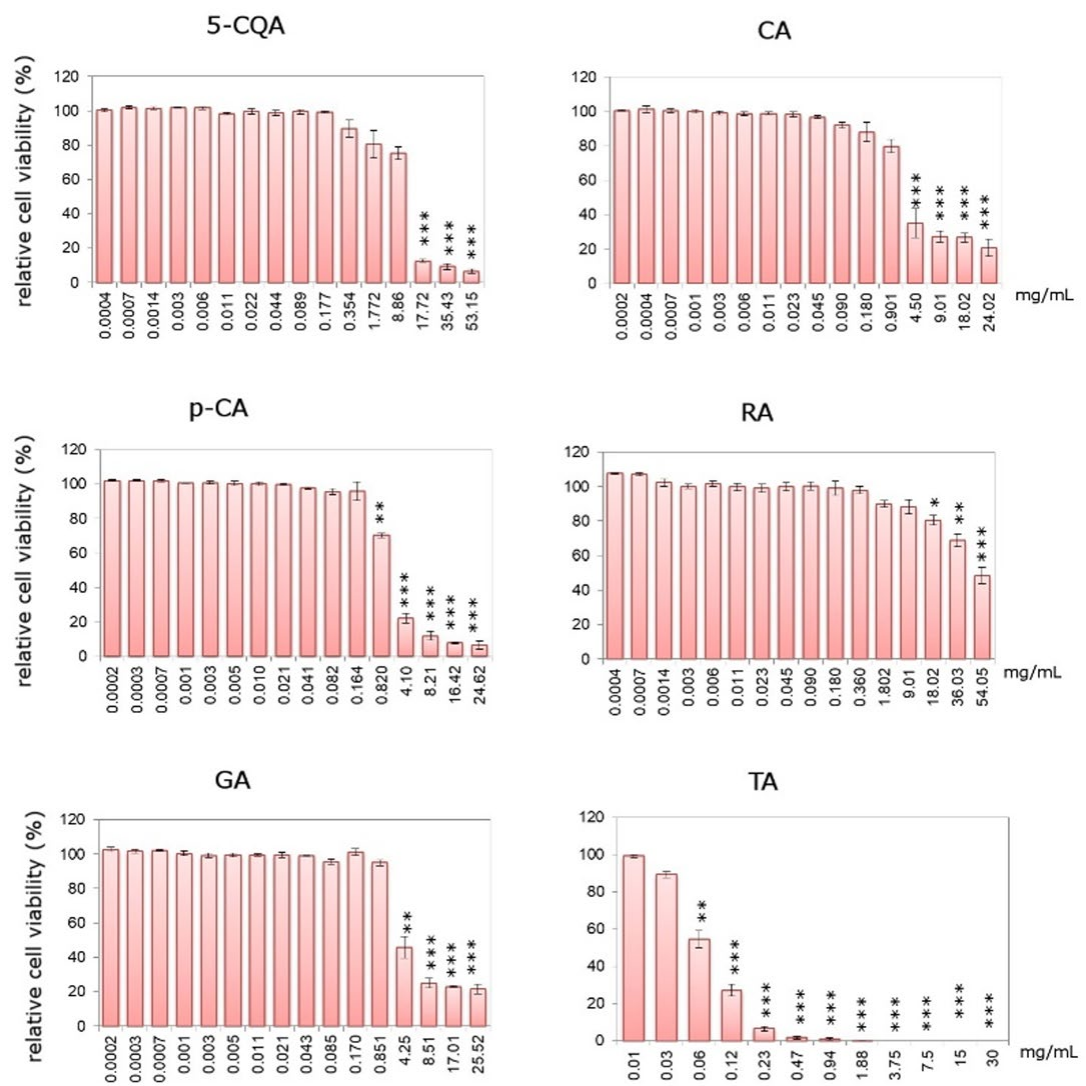
Differences in relative cell viability of *S. aureus* after 24 h of various concentrations of 5-CQA, CA, p-CA, RA, GA and TA (^x^- *p* < 0.05, ^xx^- *p* < 0.01 represent significant effects between treatments and control followed by Dunnett’s test, error bars represent the standard deviation ± SD).

**Fig. 12:**
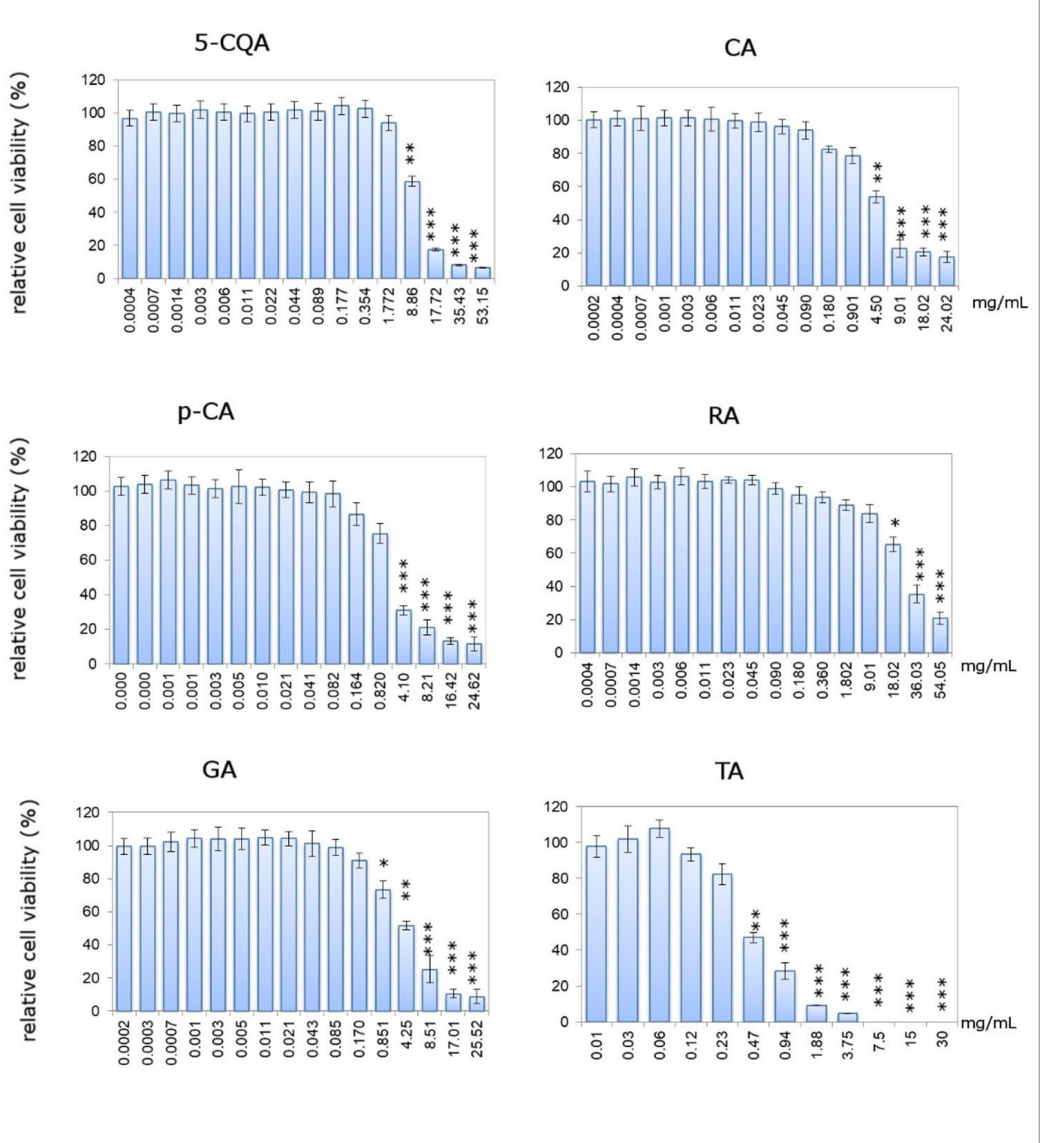
Differences in relative cell viability of *C. albicans* after 24 h of various concentrations of 5-CQA, CA, *p*-CA, RA, GA and TA (^x^- *p* < 0.05, ^xx^- *p* < 0.01 represent significant effects between treatments and control followed by Dunnett’s test, error bars represent the standard deviation ± SD).

As shown in Figs. 11 and 12; Table 1 the antimicrobial effect of the tested compounds depended on the compound concentration and the type of organism. In the case of *S. aureus* (Fig. 11), the highest antimicrobial effects were obtained with TA. The inhibitory effect of TA was observed at concentrations ranging from 0.06 mg/mL to 30 mg/mL and cell viability ranged from 54% to 0%, respectively. The MIC value was 0.23 mg/mL while MBC 3.75 mg/mL. For CA, *p*-CA and GA acids, the growth inhibitory effect on *S. aureus* was observed for concentrations higher than 4.50 mg/mL, 4.10 mg/mL and 4.25 mg/mL, respectively (MIC value was higher than 4000 mg/mL), while for 5-CQA above 17.72 mg/mL (MIC – 17.72 mg/mL). In all the mentioned treatments, the relative viability was lower than 50%. The lowest inhibition of *S. aureus* growth was observed after RA application, in which the relative viability at a concentration of 54.05 mg/mL was 48% and the MIC value was > 54.05 mg/mL. The MIC value obtained in our study is broadly consistent with findings reported by other researchers, although some variability in these values exists in literature. Reported MIC levels for TA against *S. aureus* range from 32 to 128 µg/mL^102^, 40–64 µg/mL^103,104^, and up to 0.625 mg/mL^105^. Specifically, Rashidipour et al.^106^ reported that the *S. aureus* strain exhibited resistance to TA, with minimum inhibitory concentrations (MIC) exceeding 5000 µg/mL This superior efficacy of TA compared to other tested phenolic acids is likely due to its unique structure, characterized by a significantly higher density of hydroxyl groups. As a complex polyphenol, TA can engage in multifaceted interactions with bacterial cells, including the denaturation of cell surface proteins, complexation with the cell wall, and disruption of membrane integrity^107^. The effect of antimicrobial agents may be due to the structure of the microorganism. Gram-positive bacteria are characterized by a relatively high sensitivity to antibiotics as well as antimicrobial agents. This is related to their structure and, in particular, the lack of an outer membrane that provides a protective barrier against the penetration of toxic substances and stabilizes the cell structure^108^. In our study, only TA had an inhibitory effect on the growth of *S. aureus* at lower concentrations.

Figure 12; Table 1 show the influence of CA, *p*-CA, RA, 5-CQA, GA and TA on *C. albicans*. No significant effect was observed after application of studied compounds (except TA) on tested microbial strains in the range of 0.001 do 1.0 mM. The highest inhibitory activity against *C. albicans* was observed in case of TA at concentrations higher than 3.75 mg/mL, for which the relative cells viability ranged from 47% to 0%. The MIC value was 1.88 mg/mL while MFC was 7.5 mg/mL. In a study by Rashidipour et al.^106^, the MIC for tannic acid against C. albicans was determined to be 2.5 mg/mL, which is comparable to the value obtained in this study. In case of 5-CQA, CA, *p*-CA and GA acids, the growth inhibitory effect on *C. albicans* was observed for concentrations higher than 4.00 mg/mL. The MIC values obtained for 5-CQA and p-CA were approx. 35.43 mg/mL and 18.02 mg/mL, respectively for *C. albicans*. Conversely, RA and CA were the least effective, failing to achieve complete eradication within the tested range (MIC/MFC > 54.05 mg/mL and 24.02 mg/mL, respectively). As Sung and Lee^98^ suggested, *C. albicans* is an opportunistic as well as commensal pathogen. The authors also reported that phenolic compounds (e.g. 5-CQA) may interfere with membrane potential by damaging membranes in the *C. albicans* cell, which may result in growth inhibition of the fungus. In our study, growth inhibition of *C. albicans* was observed at concentrations higher than 25 mM which may be explained by disruption of cell membranes and entry of compounds into the cell.

**Table 1:** Summary of MIC and MBC/MFC Values (mM) evaluated for *C. albicans* and *S. aureus*.

Compound	C. albicans		S. aureus	
	MIC (mg/mL)	MFC (mg/mL)	MIC (mg/mL)	MBC (mg/mL)
**5-CQA**	35.43	> 53.15	17.72	35.43
**CA**	> 24.02	> 24.02	> 24.02	> 24.02
**p-CA**	8.21	24.62	4.10	16.42
**RA**	> 54.05	> 54.05	> 54.05	> 54.05
**GA**	17.01	> 25.52	17.01	> 25.52
**TA**	1.88	7.5	0.23	3.75
**Positive control**	Itraconazole (10 µg/mL)		Gentamycin (10 µg/mL)	

## Conclusions

The study demonstrated that the antioxidant, pro-oxidant, cytotoxic and antimicrobial properties of chlorogenic, caffeic, *p*-coumaric, rosmarinic, gallic and tannic acids are strongly determined by their chemical structure, lipophilicity, and the mechanisms of radical scavenging or pro-oxidative activity. The main advance of this work lies in the integrated structure–activity relationship (SAR) and mechanistic interpretation across aqueous and lipid antioxidant assays, pro-oxidant kinetics, cellular redox markers, cytotoxic selectivity, and antimicrobial outcomes, providing a comprehensive view of how structural features translate into biological effects.

In the antioxidant assays (DPPH, ABTS, FRAP, CUPRAC, lipid peroxidation inhibition), the most potent compounds were tannic and rosmarinic acids, which consistently exhibited high radical scavenging activity, strong reducing power, and effective inhibition of lipid peroxidation. Their activity results from the abundance and arrangement of hydroxyl groups that facilitate both hydrogen atom transfer (HAT) and single electron transfer (SET), as well as from favorable lipophilicity. Tannic acid, a hydrolysable tannin containing ten galloyl groups and showing high logP (4.84), combined excellent radical scavenging with strong activity in lipid systems, while rosmarinic acid (logP = 1.60) effectively operated in both aqueous and lipid phases. Gallic acid, despite its high electron-donating capacity and strong performance in DPPH, ABTS, FRAP and CUPRAC assays, showed limited protection against lipid peroxidation due to its hydrophilic character (logP = 0.70). By contrast, *p*-coumaric acid, with only one hydroxyl group, consistently exhibited the weakest antioxidant performance. Structural complexity also influenced activity: chlorogenic acid, as an ester of caffeic acid with quinic acid, was less efficient than caffeic acid itself, due to reduced accessibility of reactive hydroxyl groups. Overall, the results demonstrate that antioxidant potential reflects a balance of hydroxyl group number, their position in the molecule, and lipophilicity, which determines the ability to act in lipid environments.

The assessment of pro-oxidant activity further highlighted structural determinants. Rosmarinic and tannic acids exhibited a rapid but transient pro-oxidant effect, with strong activity in the first 10–20 min followed by decline, likely due to the stabilization of phenoxyl radicals into less reactive quinones. In contrast, caffeic and chlorogenic acids displayed a delayed, but progressive increase in pro-oxidant potential, associated with the persistence of their phenoxyl radicals capable of further redox cycling. Gallic and *p*-coumaric acids maintained relatively constant, low pro-oxidant activity throughout the experiment, reflecting a balance between their radical reactivity and stabilization. These findings demonstrate that pro-oxidant capacity depends not only on hydroxyl group content, but also on the kinetics of radical stabilization and on the structural context revealed in antioxidant assays.

Cytotoxicity studies on melanoma A-375 cells and normal fibroblasts confirmed differential effects of the tested compounds. Tannic and gallic acids exerted the strongest cytotoxicity toward melanoma cells, reducing their viability at micromolar concentrations, while showing less pronounced effects on fibroblasts. The mechanism of this selective activity is supported by oxidative stress parameters: in A-375 cells, exposure led to increased intracellular ROS, lowered GSH/GSSG ratio, depletion of thiol groups, and elevated TBARS, reflecting redox imbalance and lipid peroxidation. In fibroblasts, these effects were less pronounced or absent, suggesting higher tolerance of normal cells. Rosmarinic acid showed moderate cytotoxicity, while caffeic and chlorogenic acids displayed concentration-dependent and sometimes biphasic effects, consistent with both antioxidant and pro-oxidant influences. *p*-Coumaric acid exhibited the weakest cytotoxic action, in line with its limited redox activity.

Antimicrobial studies revealed tannic acid as the most active agent, inhibiting the growth of *S. aureus* and *C. albicans* at relatively low concentrations. The other compounds required much higher doses to achieve significant antimicrobial effects, with rosmarinic acid being the least effective against *S. aureus*. The antimicrobial properties correlate with antioxidant and pro-oxidant behavior: tannic acid, combining strong radical scavenging with transient pro-oxidant activity, may damage microbial membranes through oxidative imbalance and direct interaction with membrane proteins and lipids, facilitated by its high lipophilicity. Gallic acid, though highly antioxidant, showed weaker antimicrobial effects, which can be explained by its hydrophilicity and limited membrane penetration. The modest activity of caffeic and chlorogenic acids likely reflects their moderate pro-oxidant potential and partial lipophilicity, while *p*-coumaric acid remained the least effective, consistent with its generally weak redox performance.

Altogether, the results indicate that the biological effects of phenolic compounds are governed by an interplay of structural factors - the number and arrangement of hydroxyl groups, degree of esterification, and lipophilicity - which determine their ability to act as antioxidants or pro-oxidants, to selectively target cancer cells through redox imbalance, and to disrupt microbial viability. These findings provide a mechanistic basis for the diverse applications of plant-derived phenolic acids as natural agents with antioxidant, anticancer and antimicrobial potential.

## Electronic Supplementary Material

Below is the link to the electronic supplementary material.

Supplementary Material 1

## Data Availability

The data presented in this study are available on request from the corresponding author.
