# Supplementary material for: Antioxidant, pro-oxidant, cytotoxic and antimicrobial properties of selected plant phenolic compounds and resulting structure-activity relationships
Source: Sci Rep. 2026 May 12;16:21589. doi: 10.1038/s41598-026-50996-z (PMC13350958; doi:10.1038/s41598-026-50996-z)
Supplement: Supplementary file 1 — Supplementary Material 1 [file 41598_2026_50996_MOESM1_ESM.docx]

**Supplementary**

**Antioxidant, pro-oxidant, cytotoxic and antimicrobial properties of selected plant phenolic compounds and resulting structure-activity relationships**

**Monika Kalinowska*^1,2^, Ewelina Gołębiewska^1^, Małgorzata Zawadzka^1^, Elżbieta Wołejko^1^, Agata Jabłońska-Trypuć^1^, Urszula Wydro^1^, Grzegorz Świderski^1^, Renata Świsłocka^1^, Waldemar Priebe^3^, Włodzimierz Lewandowski^1^**

^1^Department of Chemistry, Biology and Biotechnology, Institute of Civil Engineering and Energetics, Faculty of Civil Engineering and Environmental Science, Bialystok University of Technology, Wiejska 45E Street, 15-351 Bialystok, Poland

^2^natureTECH Centre for Natural Product Research, Bialystok University of Technology, Wiejska 45E Street, 15-351 Bialystok, Poland

^3^Department of Experimental Therapeutics, The University of Texas MD Anderson Cancer Center, 1901 East Rd., Houston, TX 77054, USA

*corresponding: [m.kalinowska@pb.edu.pl](mailto:m.kalinowska@pb.edu.pl)

**Table S1.** The content and determination methodology of the tested compounds in different natural sources.

| **Compound** | **Natural source** | **Content [mg/g d.w.]** | **Methodology** | | **Reference** |
| --- | --- | --- | --- | --- | --- |
|  |  |  | **Extraction** | **Content determination** |  |
| Chlorogenic acid | Chicory | 1.23 | Extraction (0.5 g material/3mL water + 7 mL ethanol 99.9% in 10 ml test tubes), centrifugation (eluate/50 mL water) | HPLC analysis: C18 column, 150 mm × 4.6 mm, 100A, 3˚µm, binary gradient elution (A - water/methanol (95:5 v/v)  containing 1 mL/L 99.0% formic acid, B - methanol/water (60:40 v/v) containing 1 mL/L 99.0% formic acid), flow rate 0.4 mL/min | [103] |
|  | Artichoke | 2.90 |  |  |  |
|  | Lovage | 3.25 |  |  |  |
|  | Blueberry | 1.07 |  |  |  |
|  | Chokeberry | 3.97 |  |  |  |
|  | Tomato | 0.052 | Homogenization, cooling (-18℃), 150 mg of sample/3 mL methanol:water 80:20 v/v, 30 min in rotary shaker, filtering | HPLC analysis: C18 column (100 mm 2.1 mm, 1.8 lm particle size), 100 µL extract/400 µL mobile  phase (50:50 v/v A:B), gradient elution (A – methanol, B - aqueous solution of ammonium acetate 30 mM, adjusted to pH 5 with formic acid), flow rate 0.2 ml/min | [104] |
|  | Green coffee | 57.2 – 102 | Grounding, defatting (petrol-ether), extraction (400 mL boiling UHQ-water, 15 min, stirring) | 10 mL extract diluted (1:10) with UHQ-water, determined in photometer (324 nm, against water), then added 2 mL potassium-acetate and 10 mL lead-acetate, 5 min incubation (stirring, water bath), cooling, stirring (60 min), filtering, again photometer (324 nm) | [105] |
|  | Dil | 73.61 | Soxhlet extraction (10 g fresh plant exhaustively extracted with petroleum ether followed by 95% ethanol), filtering, evaporating (rotary evaporator | RP-HPLC analysis: 50 mg/1mL methanol, filtering (0.45 µm), Inertsil® ODS-3 5 µm C18 column (4.6 X 250 mm) and ReproSil®-Pur ODS-3 C18 guard column (4.0 X 10 mm), isocratic elution (A - 0.2% phosphoric acid, pH 1.46, B – methanol), flow rate 1.2 mL/min | [4] |
|  | *Coffea canephora* | 78.43 |  |  |  |
|  | *Coffea arabica* | 59.67 |  |  |  |
|  | Leaf celery | 25.74 |  |  |  |
|  | Estragon | 52.53 |  |  |  |
| Caffeic acid | Apple | 0.0943 (*Gloster* variety) – 0.4723 (*Montuan* variety) | Dehydration of apple slices (40℃), groundind, ultrasound assisted extraction (0.5 g apple powder, 10 mL acidified methanol 80%, 30 min, 40℃, ultrasonic bath, centrifugation, filtration | RP-UHPLC-DAD analysis: Accuacore PFP (penta-fluorophenyl)  column (100 mm × 2.1 mm, 2.6 µm), gradient elution (A - water  + 0.1% formic acid, B – acetonitrile + 0.1% formic acid) | [106] |
|  | *Sanghuangporus vanini* | 96.11 | Drying, grounding, extraction (20 g powder/600 mL 95% ethanol, stirring, 30 min), sonification (450 W, 30℃, 15 min), centrifugation (5000 rpm, 10 min), vacuum rotary evaporator, lyophilization | UPLC analysis: 50 µg/300 µL methanol + 5 µL DL-4-chlorophenylalanine (1.0 mg/L), shaking, centrifugation, Waters HSS T3 column (50 mm × 2.1 mm, 1.8 μm), gradient elution (A – acetic acid 0.1%, B – acetonitrile + 0.1% acetic  acid), flow rate 0.3 mL/min | [107] |
|  | *C. canephora* | 12.33 | Soxhlet extraction (10 g fresh plant exhaustively extracted with petroleum ether followed by 95% ethanol), filtering, evaporating (rotary evaporator | RP-HPLC analysis: 50 mg/1mL methanol, filtering (0.45 µm), Inertsil® ODS-3 5 µm C18 column (4.6 X 250 mm) and ReproSil®-Pur ODS-3 C18 guard column (4.0 X 10 mm), isocratic elution (A - 0.2% phosphoric acid, pH 1.46, B – methanol), flow rate 1.2 mL/min | [4] |
|  | *C. arabica* | 3.80 |  |  |  |
|  | Pomegranate | 3.05 – 3.63 |  |  |  |
|  | Mango | 10.08 |  |  |  |
|  | Dil | 1.84 |  |  |  |
|  | Lettuce | 2.58 |  |  |  |
|  | Papaya | 5.08 |  |  |  |
|  | Castor bean | 3.52 |  |  |  |
|  | Oregano | 4.10 |  |  |  |
| *p*-coumaric acid | Apple | 0.0109 (*Montuan* variety) – 0.1631 (*Cretesc* variety) | Dehydration of apple slices (40℃), groundind, ultrasound assisted extraction (0.5 g apple powder, 10 mL acidified methanol 80%, 30 min, 40℃, ultrasonic bath, centrifugation, filtration | RP-UHPLC-DAD analysis: Accuacore PFP (penta-fluorophenyl)  column (100 mm × 2.1 mm, 2.6 µm), gradient elution (A - water  + 0.1% formic acid, B – acetonitrile + 0.1% formic acid) | [106] |
|  | Wheat straw | 6.60 | Grounding, drying (55℃, 16 h) dewaxing (toluene/ethanol 2:1 v/v, Soxhlet, 6h), 0.025 g saponified at 170℃, 2h, 7 mL 4 M NaOH, filtering, washing, adjusting pH = 2 (6 M HCl), extraction (3 x 30 mL chloroform), drying (reduced pressure, 40℃) | HPLC analysis: final residue redissolved in 2 mL methanol, Hichrom  H5ODS (250 × 4.6 mm), linear gradient elution (A - water/methanol/  acetic acid 89:10:1, B – methanol/water/acetic acid  90:9:1), flow rate 1mL/min | [108] |
|  | Rice straw | 8.60 |  |  |  |
|  | Broccoli | 0.0025 (1)  0.003 (2) | Maceration (blender), extraction (2 g/ 3x25 mL hot 70% methanol, heating, 5 min, stirring), filtering, drying (reduced pressure, 40℃), sonification (20 mL 2 M NaOH with 10 nM Na_2_H_2_EDTA and 1% ascorbic acid, 30 min, 30℃), adjusting pH = 2-3 (10 M HCl), centrifugation (12000 g, 5 min), phenolics isolation (SPE, C18, vacuum manifold, eluent – 2 mL methanol), ethyl acetate extraction (3 x 30 mL ethyl acetate), drying | Residue was re-dissolved in 2 mL of methanol  (1) CE analysis: uncoated fused silica capillaries of length 60 cm x 50 µm x 52 cm, 30℃, +30 kV, pressure injection at 50 mbar for 5 s, preconditioning: 0.1 M  NaOH (5 min), water (5 min), buffer (5 min)  (2) HPLC analysis: Luna 5 µm C18, 250 x 4.6 mm column, linear gradient elution (A – 5% v/v formic acid/water, B – methanol), flow rate 1 mL/min | [109] |
|  | Broccolini | 0.0054 (1)  0.0059 (2) |  |  |  |
|  | Brussels sprout | 0.0047 (1)  0.0042 (2) |  |  |  |
|  | Cabbage | 0.0027 (1)  0.0024 (2) |  |  |  |
|  | Cauliflower | 0.0027 (1)  0.0023 (2) |  |  |  |
| Rosmarinic acid | Parsley | 91.53 | Soxhlet extraction (10 g fresh plant exhaustively extracted with petroleum ether followed by 95% ethanol), filtering, evaporating (rotary evaporator | RP-HPLC analysis: 50 mg/1mL methanol, filtering (0.45 µm), Inertsil® ODS-3 5 µm C18 column (4.6 X 250 mm) and ReproSil®-Pur ODS-3 C18 guard column (4.0 X 10 mm), isocratic elution (A - 0.2% phosphoric acid, pH 1.46, B – methanol), flow rate 1.2 mL/min | [4] |
|  | Estragon | 53.59 |  |  |  |
|  | Water spinach | 83.03 |  |  |  |
|  | Lemon balm | 199.08 |  |  |  |
|  | Marjoram | 79.54 |  |  |  |
|  | Oregano | 99.02 |  |  |  |
|  | Lemon thyme | 101.76 |  |  |  |
|  | Rosemary | 26.11 |  |  |  |
|  | *C. canephora* | 16.63 |  |  |  |
| Gallic acid | *Sanghuangporus vanini* | 712.84 | Drying, grounding, extraction (20 g powder/600 mL 95% ethanol, stirring, 30 min), sonification (450 W, 30℃, 15 min), centrifugation (5000 rpm, 10 min), vacuum rotary evaporator, lyophilization | UPLC analysis: 50 µg/300 µL methanol + 5 µL DL-4-chlorophenylalanine (1.0 mg/L), shaking, centrifugation, Waters HSS T3 column (50 mm × 2.1 mm, 1.8 μm), gradient elution (A – acetic acid 0.1%, B – acetonitrile + 0.1% acetic  acid), flow rate 0.3 mL/min | [107] |
|  | Java plum | 35.90 | Batch extraction (50 mL glass reactor with three bladed glass turbines for stirring, 20 mL water, 105 min, 250 rpm, 50℃, centrifugation, filtering) | HPLC analysis: 10-times dilution, InertClone column (5 μ x 4.6 mm x 250 mm), isocratic elution (methanol-acetonitrile-water 10:10:80), flow rate 1 mL/min | [110] |
|  |  | 54.50 | Ultrasound assisted extraction (22 and 40 kHz, 215 W, water (1:15 ratio), cylindrical flat bottom glass vessel, 12 min, 35℃, centrifugation, filtering) |  |  |
|  | *Ficus auriculata* | 9.885 (methanol)  8.526 (water pH 8) | Drying leaves (50℃ overnight, grinding, ultrasound assisted extraction (sonication bath, 37 kHz, methanol 50% or water 8 pH, 15 min, 50℃), centrifugation (10000 rpm, 10 min), filtering | HPLC analysis: Shimpack  C18 column (250 mm × 4.6 mm), isocratic elution (A – acetonitrile, B – 0.1% orthophosphoric acid), flow rate 0.8 mL/min | [111] |
|  | White tea | 0.27 | Ultrasound assisted extraction (0.50 g dried tea powder, 2 x 10 mL methanol 70% (v/v), ultrasonic cleaning bath, 10 min, room temperature), centrifugation (1000 rpm, 5 min), filtering | UPLC analysis: a Waters ACQUITY UPLC® HSS,C18 column (150 × 2.1 mm  i.d., 1.8 μm), A – acetonitrile, B – acidified water (0.1% formic acid). | [5] |
|  | Black Tea | 0.76 |  |  |  |
|  | Apple | 0.0049 (*Richard* variety) – 0.2174 (*Spartan* variety) | Dehydration of apple slices (40℃), grounding, ultrasound assisted extraction (0.5 g apple powder, 10 mL acidified methanol 80%, 30 min, 40℃, ultrasonic bath, centrifugation, filtration | RP-UHPLC-DAD analysis: Accuacore PFP (penta-fluorophenyl)  column (100 mm × 2.1 mm, 2.6 µm), gradient elution (A - water  + 0.1% formic acid, B – acetonitrile + 0.1% formic acid) | [106] |
| Tannic acid | *Quercus infectoria* subsp. *boissieri* and subsp. *infectoria* | 127.683 (80% methanol)  67.200 (70% acetone)  81.012 (96% ethanol) | Maceration (10 g/200 ml methanol 80% or acetone 70%), 8 hours, room temp.), filtering, rotary evaporator  Homogenization (2 g plant sample, 96% ethanol), stored over night (45℃), centrifugation (4000 rpm), rotary evaporator | HPLC analysis: Zorbax Eclipse XDB-C18 column (250  x 4.6 mm x 5 µm), gradient elution (A – formic acid 3%, B – methanol), flow rate 0.8 mL/min | [112] |
|  | Grape seeds | 41.00 | Soxhlet extraction (30 g/200 ml ethanol, 40-60℃, 24 h), vacuum rotary evaporator, oil separation (100 ml water, separation funnel, ethyl acetate, filtration), | DMCA (4-Dimethylamino-cinnam aldehyde) (1 mL extract + 5 mL DMCA, 640 nm) | [113] |
|  | Pomegranate peels | 276.00 | Soxhlet extraction (20 g powder/200 ml water, 100℃, 24 h), ethyl acetate, separation funnel, rotary evaporator |  |  |

**Figure S1.** UV/Vis spectra of 5-CQA, CA, *p*-CA, RA, GA (C= 0.05 mM) and TA* (C= 0.5 mM) in methanol recorded in the range of 190-390 nm.
